# Effectiveness of a Text Message Intervention Promoting Seat Belt Use Among Young Adults: A Randomized Clinical Trial

**DOI:** 10.1001/jamanetworkopen.2022.31616

**Published:** 2022-09-21

**Authors:** Brian Suffoletto, Maria L. Pacella-LaBarbara, James Huber, M. Kit Delgado, Catherine McDonald

**Affiliations:** 1 Department of Emergency Medicine, Stanford University, Palo Alto, California; 2 Department of Emergency Medicine, University of Pittsburgh, Pittsburgh, Pennsylvania; 3 University of West Virginia School of Medicine, Morgantown; 4 Department of Family & Community Health, University of Pennsylvania School of Nursing, Philadelphia; 5 PENN Injury Science Center, University of Pennsylvania, Philadelphia

## Abstract

**Importance:**

Approximately 1 in 10 adults do not always wear a seat belt, with the lowest use rates reported among young adults.

**Objective:**

To determine the efficacy of a 6-week automated behavioral text message program promoting seat belt use compared with an attention control.

**Design, Setting, and Participants:**

This parallel, 2-group, single-blind, individually randomized clinical trial included a convenience sample of patients recruited from 4 emergency departments in 2 cities in Pennsylvania from December 2019 to September 2021, with follow-ups at 6 and 12 weeks after randomization. Patients in stable condition aged 18 to 25 years who, in standardized screening, reported driving or being a passenger in a car without always using a seat belt in the past 2 weeks were eligible for recruitment. Participants who completed a 2-week trial run-in phase were randomly assigned 1:1 to the intervention or the assessment control. Data were analyzed from October 2019 to January 2020.

**Interventions:**

The intervention group received Safe Vehicle Engagement (SAVE), a 6-week automated interactive text message program, including weekly seat belt use queries with feedback and goal support to promote consistent use of a seat belt. The control group received identical weekly seat belt use queries but no additional feedback.

**Main Outcomes and Measures:**

The primary outcome was the proportion of young adults reporting always wearing a seat belt over the past 2 weeks, collected at 6 weeks (after a 2-week run-in) via web-based self-assessments and analyzed under intent-to-treat models using multiple imputation procedures. Sensitivity analyses included complete-case analyses of ordered categorical outcomes by vehicle seat position. Secondary outcomes included seatbelt use at 12 weeks and select cognitive constructs related to seat belt use.

**Results:**

A total of 218 participants (mean [SD] age, 21.5 [2.1] years; 139 [63.8%] women) were randomized, with 110 randomized to SAVE and 108 randomized to the control group. A total of 158 individuals (72.4%) were included in the 6-week follow-up. The rate of always using a seat belt over the past 2 weeks at the 6-week follow-up was 41.3% (95% CI, 30.6%-52.0%) among SAVE participants and 20.0% (95% CI, 10.6%-29.3%) among control participants (odds ratio [OR], 2.8; 95% CI, 1.4-5.8; *P* = .005). A total of 140 individuals (64.2%) participated in the 12-week follow-up. At 12 weeks, the rate of always using a seat belt over the past 2-weeks was 42.8% (95% CI, 31.2%-54.2%) among SAVE participants and 30.7% (95% CI, 19.6%-41.6%) among control participants (OR, 1.7; 95% CI, 0.9-3.4; *P* = .13). When examining ordered categories of seat belt use by seat position, there were significantly greater odds of wearing a seat belt at 6 and 12 weeks among SAVE participants vs control participants (eg, 6 weeks for driver: OR, 5.2; 95% CI, 2.6-10.5; 6 weeks for front passenger: OR, 4.3; 95% CI, 2.2-8.2; 6 weeks for back passenger: OR, 4.3; 95% CI, 2.2-8.2).

**Conclusions and Relevance:**

In this randomized clinical trial, an interactive text message intervention was more effective at promoting seat belt use among targeted young adults than an attention control at 6 weeks. There was no significant difference between groups in always wearing a seat belt at 12 weeks. These findings, if replicated in a larger sample, suggest a scalable approach to improve seat belt use.

**Trial Registration:**

ClinicalTrials.gov Identifier: NCT03833713

## Key Points

**Question:**

What is the effect of a 6-week text-message program on seat belt use among young adults?

**Findings:**

In this randomized clinical trial that included 218 young adults who reported not always using a seat belt over the past 2 weeks at baseline, the proportion reporting always wearing a seat belt at 6 weeks was 41% in the intervention group vs 20% in the assessment control group, a significant difference.

**Meaning:**

These findings suggest that for young adults at risk of not using a seat belt, the use of an automated 6-week text message program increased short-term prevalence of seat belt use.

## Teaser

This randomized clinical trial assesses the efficacy of an automated behavioral text message program promoting seat belt use among young adults compared with an attention control.

## Introduction

In 2020, the US Department of Transportation National Highway Traffic Safety Administration estimated that 38 680 people died in motor vehicle crashes (MVCs), with more than half of all MVC fatalities involving drivers or passengers not wearing seat belt restraints.^1^ Despite solid evidence that seat belt use can reduce risk of major injury^2^ and save lives,^3^ estimates from 2020 suggest that an estimated 10% of vehicle occupants still do not wear seat belts.^4^ Young adult drivers and passengers aged 18 to 24 years have the highest MCV-related nonfatal injury rates of all adults and relatively low rates of seat belt use compared with other age ranges.^5^

Evidence-based prevention programs to increase seat belt use among targeted groups of young adults are needed to reduce injuries and prevent deaths. Brief in-person behavioral interventions targeting vehicle safety have shown to improve seat belt use among older adults^6^ but have not been designed to meet the unique needs of young adults or implemented broadly to affect public health. Mobile digital behavioral interventions offer advantages to in-person behavioral interventions in their portability and automation^7^ and have shown positive effects on influencing other risk behaviors among young adults^8^ but have not yet been evaluated for improving seat belt use.

Extending our prior work developing effective text message interventions to reduce alcohol use and texting while driving in young adults,^9,10^ we designed an automated text message-based behavioral intervention, Safe Vehicle Engagement (SAVE), focused on promoting seat belt use. It targets key constructs of the Theory of Planned Behavior,^11^ attempting to alter attitudes toward wearing a seatbelt, providing cues to action, and boosting self-efficacy. Consistent with self-regulation^12^ and goal setting^13^ theories, SAVE incorporates weekly check-ins of seat belt use with tailored feedback as well as goal commitment prompts for the coming week, feedback, and reminders. The primary hypothesis was that participants in the SAVE group would be more likely to report seat belt use at 6 weeks after randomization than participants in the assessment control group.

## Methods

This randomized clinical trial was approved by the University of Pittsburgh and University of Pennsylvania institutional review boards. All participants provided written informed consent. This study is reported according to the Consolidated Standards of Reporting Trials (CONSORT) reporting guideline.

### Trial Design

The study was a parallel 2-group individually randomized clinical trial among young adults with inconsistent seat belt use. We chose to recruit from the emergency department (ED) since it may be, for many young adults, their only point of intersection with health care.^14^ This study compared an interactive text message intervention to promote consistent use of a seat belt (ie, SAVE) with a text message–based assessment control. Assessor-blinded self-reported outcomes were assessed at 6 and 12 weeks after randomization. The study was prespecified in the trial protocol, available in Supplement 1.

### Recruitment and Enrollment

**Figure 1.**
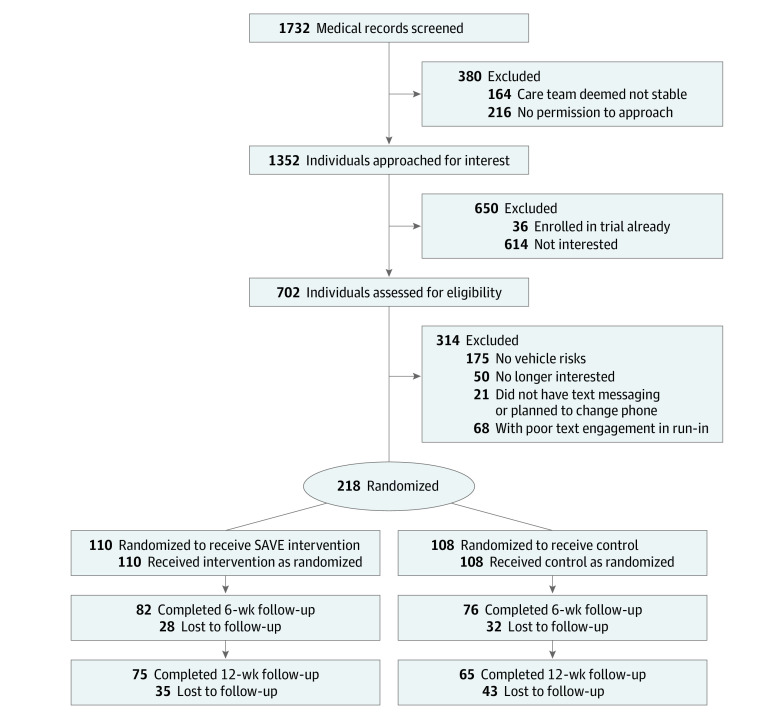
Participant Recruitment Flowchart SAVE indicates Safe Vehicle Engagement.

Between December 3, 2019, and June 18, 2021, with the exception of April to August 2020 (due to COVID-19 restrictions), 1732 patients aged 18 to 25 years who presented to 1 of 4 participating EDs in Pennsylvania and who spoke English were identified through medical record screening, and 1352 individuals who were medically stable were approached for screening. Among these, 702 individuals were assessed for eligibility. The inclusion criterion was reporting inconsistent (ie, less than always) seat belt use in the past 2 weeks either as a driver or passenger. Patients were excluded if they reported not owning a personal mobile phone with text messaging, planned to change their phones in the next 3 months, or had no plan to drive or ride in a vehicle in the next month. Recruitment occurred at times and on days when a research associate was available, providing a convenience sampling of screened patients. Details of the recruitment flow are depicted in Figure 1.

### Run-in and Randomization

A total of 286 participants completed written informed consent and the baseline assessment and were instructed to text a study telephone number within the next 24 hours. Once this match was recognized, participants received several texts welcoming them to the study and describing the trial run-in phase. Participants were instructed that they could drop out of the SAVE program at any time by texting *stop.* Each Sunday at 4 pm, participants received the text message: “How often have you been a passenger or driver in a car the past week? 0=never; 1=a few times; 2=most days; 3=every day,” and if they responded with a value 1, 2, or 3, received: “How often did you wear a seat belt? 0=never; 1=a few times; 2=most of the time; 3=every time.” Participants who responded to at least 50% of these queries over the 2-week run-in phase were randomized to SAVE or control groups by a computer algorithm that automated random allocation in a 1:1 sequence. Random assignments were in blocks of 4 based on recruitment site and concealed from participants and research staff throughout the trial.

### The SAVE Intervention

Communication within SAVE is grounded in best practices for digital behavioral interventions,^15^ including personalization of each weekly dialogue with the participant’s name and identification of message origin as *The SAVE Team*. Each message was drafted by a team of health behavioral scientists and written at an appropriate level of literacy (Flesch-Kincaid^16^ grade 6 readability). SAVE software was run by the Office of Academic Computing at the University of Pittsburgh Medical Center.

On the day of randomization, participants allocated to SAVE received a series of welcome messages describing what to expect over the intervention period. Participants received 2 weekly queries, which were identical to those sent in the run-in, and each Sunday, if a participant reported wearing a seat belt every time, they received a positive reinforcement message. If they reported less than every time seat belt use, they received a feedback message reframing the goal failure as an opportunity for a fresh start.^17^ Independent of their past week report, participants received a goal commitment query to wear a seat belt that week. If a participant agreed, they received a positive reinforcement message. On Wednesdays at 4 pm during weeks when an individual agreed to commit to a goal, they received a text goal reminder. If they did not agree to commit to a goal, they received a fact either about the risks of not wearing a seatbelt or benefit to wearing a seat belt.

### Assessment Control

We chose a control group that received similar attention to the intervention group to help isolate intervention feedback effects from repeated assessments. Participants allocated to the control group received weekly vehicle and seat belt queries identical to those sent in the run-in (ie, “How often have you been a passenger or driver in a car the past week?” and “How often did you wear a seat belt?”) without receiving any feedback or goal support.

### Procedures

The baseline survey was conducted in-person in the ED using a web-based questionnaire and was hosted on a secure server. At baseline, participants provided demographic information, past 2-week vehicle use and safety, driving history, and seat belt–related cognitions. Self-reported race categories included Asian, Black/African American, White/Caucasian, and other and was coded as mixed if more than 1 category was chosen. Participants did not provide further information if other race was selected. Participants could also select Hispanic ethnicity separately. Sex assigned at birth was measured given that rates and consequences of vehicle safety differ between men and women. Sex and race were measured as covariates given that seat belt use rates have been found to differ among different sex and race categories.^18^

Both groups completed text messaging for 6 weeks. For all text queries, missing responses were reprompted once. At the completion of 6 weeks, all participants received the message “This completes the SAVE text messaging. Thanks for participating.” Follow-up assessments at 6 and 12 weeks after randomization were conducted via a web-based questionnaire that required the participant to enter a unique password and hosted on a secure server. Participants who did not complete the survey online within 2 weeks were contacted over telephone by research staff blind to treatment assignment. Participants were eligible to receive a total of $45 for participation in the study, including $15 for completing the baseline assessment battery, $15 for completing the 6-week follow-up assessment, and $15 for completing the 12-week follow-up assessment. Participants were not compensated for completing text messages queries.

### Outcome Measures

#### Primary Outcome

The primary outcome measure was the prevalence of always seat belt use at 6 weeks after randomization (excluding the 2 week run-in period). Items to measure seat belt use were adapted from NHTSA’s Motor Vehicle Occupant Safety Survey.^19^ These included: “In the past 2-weeks, how often have you… driven a car?”; “…been a passenger in the front seat of a car?”; and “…been a passenger in the back seat of a car?” with response options of never, a few times, most days, and every day. For each vehicle seat position, we asked “How often did you wear a seat belt..?” with response options of never, a few times, most of the time, and always. Always seat belt use was defined as an individual reporting always to the frequency of seat belt use for all vehicle positions, coded as 1 if always was reported for all seat positions or coded as 0 if never, a few times, or most of the time was reported for any seat position. For example, if an individual reported wearing a seat belt always as a driver but most of the time as a back passenger, their outcome was coded as 0. We chose this as our primary outcome because the goal of the SAVE intervention was to increase consistent seat belt use independent of seat position.

#### Secondary Outcomes

Secondary outcome measures included seat belt use at 12 weeks and select cognitive constructs related to seat belt use measured using Theory of Planned Behavior^11^ and the Health Belief Model,^20^ including perceived peer norms, perceived danger of not wearing a seat belt, and perceived control. Perceived norms were measured with the question “How often do your friends use their seat belt?” with response options never, rarely, most of the time, and always. For the purposes of analyses, we dichotomized responses to most of the time or always vs any response less than most of the time. Perceived danger was measured using “How dangerous is it to not wear a seat belt?” with response options not at all, somewhat, very, and completely. For the purposes of analyses, we dichotomized responses to completely vs any response less than completely. Perceived control was measured using “How much do you agree: I have complete control over whether I wear a seat belt.” with response options strongly disagree, disagree, somewhat agree, mostly agree, and strongly agree. For the purposes of analyses, we dichotomized responses to strongly agree vs any response less than strongly agree.

### Statistical Analysis

We first compared baseline characteristics between study groups, as well as primary and secondary outcomes using the *t* test for continuous data and χ^2^ tests for categorical data. Primary outcome analyses were based on intention-to-treat (ITT). Based on estimates from Sommers et al,^6^ the study was powered to detect a treatment difference of 15%, in which 25% of the SAVE participants and 10% of the assessment controls report always using a seat belt at the 6-week follow-up. Following current recommendations^21,22^ when between 5% and 50% of outcomes are missing, we used multiple imputation procedures. Univariate associations of selected observed variables and missing outcomes are presented in eTable 1 in Supplement 2. Because the pattern of missingness was nonmonotonic, we used multiple imputation chained equations.^23^ Because the intervention group and percentage of weeks with text message reports of seat belt use were not associated with missingness, data were assumed to be missing at random.^24^ We conditioned the estimation of missing outcome values on age, non-White race, and intervention and generated 40 imputed data sets based on the rule of thumb that the number of imputations should be at least equal to the percentage of incomplete cases.^23^ We checked how well the imputation model fits the observed outcome data by inspecting the distributions of the outcomes in each treatment group. Sensitivity of the findings to imputation were assessed by conducting completed case analyses (CCA), in which the 6-week outcome analyses only included participants who had completed the 6-week follow-up and the 12-week outcome analyses only included participants who had completed the 12-week follow-up. Sensitivity of findings to vehicle seat position and outcome definition were assessed by conducting CCAs for ordered categories of seat belt use (ie, never, a few times, most of the time, and always) for driver, front passenger, and back passenger separately using ordinal logistic regression models. Secondary analyses attempted to identify potential mechanisms of effects by CCA with cognitive constructs related to seat belt use. Estimated treatment effects are reported as odds ratios (ORs) with 95% CIs. All hypothesis tests were conducted at a 2-tailed α = .05 significance level. Analyses were conducted using Stata statistical software version 16.1 (StataCorp) from October 2019 to January 2020.

## Results

### Participant Characteristics

A total of 218 participants (mean [SD] age, 21.5 [2.1] years; 139 [63.8%] women) were randomized, with 110 randomized to SAVE and 108 randomized to control. Overall, there were 9 Asian participants (4.1%), 72 Black participants (33.0%), 13 participants (6.0%) who identified as more than 1 race, 111 White participants (50.9%), 13 participants (6.0%) participants who identified as other race; 31 participants (14.2%) reported Hispanic ethnicity. Only 3 participants (1.4%) presented with concerns related to MVCs. Table 1 presents the self-reported baseline characteristics of enrolled participants. The most common reasons for not wearing a seat belt were forgetting (116 participants [53.2%]) and finding it uncomfortable (92 participants [42.2%]). There were no significant between-group differences in any baseline variables, indicating a balanced sample.

**Table 1.** Baseline Characteristics of Randomized Participants

Characteristics	Participants, No. (%)		
	Total (N = 218)	SAVE (n = 110)	Control (n = 108)
Age, mean (SD), y	21.5 (2.1)	21.5 (2.2)	21.6 (2.0)
Assigned sex at birth			
Women	139 (63.8)	68 (61.8)	71 (65.7)
Men	79 (36.2)	42 (38.2)	37 (34.3)
Race			
Asian	9 (4.1)	7 (6.4)	2 (1.9)
Black	72 (33.0)	37 (33.6)	35 (32.4)
Mixed	13 (6.0)	5 (4.6)	8 (7.4)
White	111 (50.9)	51 (46.4)	60 (55.6)
Other^a^	13 (6.0)	10 (9.1)	3 (2.8)
Hispanic ethnicity	31 (14.2)	17 (15.5)	14 (13.0)
Emergency care chief concern category			
Motor vehicle crash	3 (1.4)	2 (1.8)	1 (0.9)
Musculoskeletal pain	112 (51.4)	62 (57.4)	50 (45.4)
Headache	57 (26.2)	20 (18.5)	37 (33.6)
Abdominal, gynecological, or urological	11 (5.1)	6 (5.6)	5 (4.6)
Chest pain, short of breath, or syncope	12 (5.5)	4 (3.7)	8 (7.3)
Other	26 (11.8)	16 (14.8)	10 (9.1)
Current college enrollment	82 (37.6)	44 (40.0)	38 (35.2)
Past year driving			
Traffic ticket as driver	34 (15.6)	13 (11.8)	21 (19.4)
Motor vehicle crash as driver	34 (14.5)	10 (9.1)	24 (22.2)
Reasons for not wearing a seat belt^b^			
Forgot	116 (53.2)	63 (57.3)	53 (49.1)
Uncomfortable	92 (42.2)	50 (45.5)	42 (38.9)
Like freedom	28 (25.9)	24 (21.8)	28 (25.9)
Do not think they help	15 (6.9)	7 (6.4)	8 (7.4)
Other	26 (11.9)	17 (15.5)	9 (8.3)

Abbreviation: SAVE, Safe Vehicle Engagement.

^a^ Includes individuals who identified as other race.

^b^ Reasons provided were not mutually exclusive.

Engagement was similar between treatment arms. SAVE participants completed a mean (SD) of 4.8 (1.6) weeks with text replies, compared with 4.9 (1.7) weeks among control participants (*t* test *P* = .65). A total of 56 SAVE participants (50.9%) completed assessments for all 6 weeks, compared with 59 control participants (54.6%; χ^2^ *P* = .58). Overall, 158 participants (72.4%) completed the 6-week follow-up and 140 participants (64.2%) completed the 12-week follow-up; 130 participants (59.6%) completed both follow-ups, 28 participants (12.8%) completed only the 6-week follow-up, 10 participants (4.6%) completed only the 12-week follow-up, and 50 participants (22.9%) did not complete either follow-up.

### Primary Outcomes

**Figure 2.**
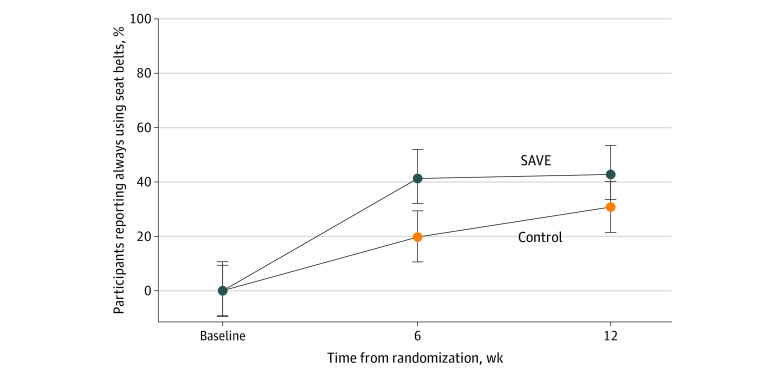
Change in Always Seat Belt Use Over Time by Treatment Condition Error bars represent 95% CIs of estimates. SAVE indicates Safe Vehicle Engagement.

At 6 weeks, the follow-up completion rate was 74.5% (95% CI, 65.4%-82.4%) among SAVE participants and 70.4% (95% CI, 60.8%-78.8%) among control participants (χ^2^ *P* = .49). At 12 weeks, the follow-up completion rate was 68.2% (95% CI, 58.6%-76.7%) among SAVE participants and 60.2% (95% CI, 50.3%-69.5%) among control participants (χ^2^ *P* = .22). Table 2 and Figure 2 show the prevalence of past 2-week always using a seat belt at 6- and 12-week follow-ups, in addition to ORs using ITT and CCA. Rates of reporting always using a seat belt over the past 2 weeks at the 6-week follow-up in the ITT model were 41.3% (95% CI, 30.6%-52.0%) among SAVE participants and 20.0% (95% CI, 10.6%-29.3%) among control participants (OR, 2.8; 95% CI, 1.4-5.8; *P* = .005). Findings at 6 weeks using the CCA model were similar to the ITT model, supporting the multiple imputation model estimates (Table 2).

**Table 2.** Participants Reporting Always Wearing a Seatbelt Under ITT and CCA at 6 and 12 Weeks

Measure	Participants, % (95% CI)		Odds ratio (95% CI)	*P* value
	SAVE	Control		
6 wk				
ITT	41.3 (30.6-52.0)	20.0 (10.6-29.3)	2.8 (1.4-5.8)	.004
CCA	41.5 (30.7-52.9)	19.7 (11.5-52.9)	2.9 (1.4-5.9)	.03
12 wk				
ITT	42.8 (31.2-54.2)	30.7 (19.6-41.6)	1.7 (0.9-3.4)	.13
CCA	42.7 (31.3-54.6)	30.8 (19.9-43.4)	1.7 (0.8-3.4)	.15

Abbreviations: CCA, complete case analysis; ITT, intention-to-treat analysis; SAVE, Safe Vehicle Engagement.

There was no difference between groups in the follow-up completion rate at 12 weeks (68.2% [95% CI, 58.6%-76.7%] of SAVE participants vs 60.2% [95% CI, 50.3%-69.5%] of control participants; *P* = .22). Rates of reporting always using a seat belt over the past 2 weeks at the 12-week follow-up under the ITT model were 42.8% (95% CI, 31.2%-54.2%) among SAVE participants and 30.7% (95% CI, 19.6%-41.6%) among control participants (OR, 1.7; 95% CI, 0.9-3.4; *P* = .13). Reported rates of always using a seatbelt at 12 weeks using the CCA model were similar to the ITT model, supporting the multiple imputation model estimates (Table 2).

### Sensitivity Analyses

We examined ordered categories of self-reported seat belt use over the past 2 weeks (ie, never, a few times, most of the time, and always) at 6- and 12-week follow-ups by location in vehicle using CCA and found significant differences across driver, front passenger, and back passenger positions between SAVE and control groups at 6 weeks (driver: OR, 5.2; 95% CI, 2.6-10.5; front passenger: OR, 4.3; 95% CI, 2.2-8.2; back passenger: OR, 4.3; 95% CI, 2.2-8.2) and 12 weeks (Table 3).

**Table 3.** Ordered Categorical Outcomes Stratified by Vehicle Seat Position

Seat position	Baseline			6 wk			12 wk		
	No. (%)		OR (95% CI)	No. (%)		OR (95% CI)	No. (%)		OR (95% CI)
	SAVE (n = 110)	Control (n = 108)		SAVE (n = 82)	Control (n = 76)		SAVE (n = 75)	Control (n = 65)	
Driver									
Participants	88 (80.0)	89 (82.4)		73 (89.0)	66 (86.8)		66 (88.0)	57 (87.7)	NA
Seat belt use									
Never	7 (8.0)	4 (4.5)	1.1 (0.7-1.9)	1 (1.4)	4 (6.1)	5.2 (2.6-10.5)	1 (1.5)	2 (3.5)	3.5 (1.7-7.4)
A few times	13 (14.8)	22 (24.7)		4 (5.5)	10 (30.3)		4 (6.1)	15 (26.3)	
Most of the time	26 (29.6)	22 (24.7)		13 (17.8)	16 (24.2)		11 (16.7)	12 (21.1)	
Always	42 (47.7)	41 (46.1)		55 (75.3)	26 (39.4)		59 (75.8)	28 (49.1)	
Front passenger									
Participants	105 (95.5)	104 (96.3)		107 (97.3)	106 (98.2)		72 (96.0)	63 (96.9)	NA
Seat belt use									
Never	6 (5.7)	11 (10.6)	1.2 (0.7-1.9)	2 (2.5)	7 (9.7)	4.3 (2.2-8.2)	4 (5.6)	5 (7.9)	2.2 (1.1-4.2)
A few times	23 (21.9)	26 (25.0)		3 (3.8)	16 (22.2)		4 (5.6)	11 (17.50)	
Most of the time	39 (37.1)	29 (27.9)		20 (25.3)	23 (31.9)		20 (27.8)	20 (31.8)	
Always	37 (35.2)	38 (36.5)		54 (68.4)	26 (36.1)		44 (61.1)	27 (42.9)	
Back passenger									
Participants	99 (90.0)	98 (90.7)		60 (73.2)	57 (75.0)		54 (72.0)	46 (70.8)	NA
Seat belt use									
Never	23 (23.2)	29 (29.6)	1.5 (0.9-2.4)	5 (8.3)	13 (22.8)	2.9 (1.5-5.6)	5 (9.3)	14 (30.4)	4.8 (2.2-10.3)
A few times	37 (37.4)	40 (40.8)		14 (23.3)	20 (35.1)		7 (13.0)	16 (34.8)	
Most of the time	36 (36.4)	26 (26.5)		20 (33.3)	14 (24.6)		17 (31.5)	7 (15.2)	
Always	3 (3.0)	3 (3.1)		21 (35.0)	10 (17.5)		25 (46.3)	9 (19.6)	

Abbreviations: NA, not applicable; OR, odds ratio; SAVE, Safe Vehicle Engagement.

### Secondary Outcomes

To explore potential mechanisms of effects of SAVE compared with controls, we examined the percentage of participants in each arm reporting perceived seat belt peer norms, perceived danger of not wearing a seat belt, and perceived control related to wearing a seat belt (eTable 2 in Supplement 2). We found that the percentage of SAVE participants reporting friends using seat belts at least most of the time increased from 59.1% (95% CI, 49.3%-68.4%) of participants at baseline to 76.5% (95% 4 CI, 65.8%-85.2%) of participants at 6 weeks and 77.0% (95% CI 65.8%-86.0%) at 12 weeks. Controls went from 55.6% (95% CI, 45.7%-65.1%) of participants at baseline to 55.4% (95% CI, 43.4%-67.0%) of participants at 6 weeks and 67.7% (95% CI, 54.9%-78.8%) of participants at 12 weeks.

## Discussion

This randomized clinical trial provides the first experimental evidence, to our knowledge, that an automated and interactive text message intervention can increase short-term seat belt use among a sample of young adults identified in the ED with inconsistent seat belt use. We found that at 6 weeks, both groups significantly increased their seat belt use, but there was a 21% greater likelihood of participants randomized to SAVE to report always wearing their seat belt at the 6-week primary end point compared with control participants. The intervention effects were durable out to 12 weeks, meaning there was not a decline in seat belt use after cessation of the intervention. Although the estimated 12% greater rate of always seat belt use in the intervention group was not statistically significant, we found significant differential effects when examining ordered categories of seat belt use.

To our knowledge, there are no prior digital behavioral interventions targeting seat belt use with which to compare our findings; however, our work builds on previous text message interventions for health promotion and harm reduction in young adults.^25^ Intervention effects in our study were larger than a study testing a combined intervention using face-to-face counseling plus telephone booster among older adult ED patients, which increased always seat belt use from 53% at baseline to 59% at 3 months compared with a contact control group who decreased always seat belt use from 53% at baseline to 50% at 3 months.^6^

A few secondary findings merit discussion. First, we found relative improvements in seat belt use in the assessment control group. We believe that this may be due to assessment reactivity related to the 6 weeks of text message queries about seat belt use. This fits with prior literature on the effects of assessment reactivity on behavior change and is common in behavioral trials.^26^ Second, there were improvements over time in intervention participants’ belief that their friends use seat belts, and these differences were significantly greater than controls. This finding fits with current understanding of the influence of peers on driving behaviors in young adults and could indicate that individuals in the intervention arm either corrected initial misperceptions of peer seat belt use, altered their friend group, or influenced their friend groups to increase seat belt use over time.

Several strengths of our study design and findings are noteworthy. First, this study included a sample with diversity across a number of demographic characteristics (ie, race, ethnicity, and education). Second, the intervention was completely automated and the feedback messages were based on decision rules that were developed prior to trial initiation, essentially eliminating the costs and uncontrolled variability that exists in delivery of in-person interventions. Third, we did not pay participants specifically to use the intervention (ie, remuneration based on number of weeks of responses). This coupled with the relatively high engagement over the 6 weeks of the intervention support our belief that the intervention could be used outside of a clinical trial.

Our findings have several important implications for public health interventions. First, based on effects at 6 weeks, approximately 5 targeted young adults need to be exposed to SAVE compared with weekly self-monitoring to prevent 1 young adult from being unrestrained in a vehicle. Given the low cost to send text messages and the automated nature of the intervention, barriers to implementation would be lower than with in-person behavioral interventions. For health care settings, access to the SAVE intervention could be provided as a digital prescription. To impact broader populations of young adult drivers, access could be provided through drivers’ insurance plans.^27^

### Limitations

This study has several limitations. The outcome measures were based on self-reported data, which may be subject to recall or social desirability biases and may have increased the apparent efficacy of the intervention. However, inclusion of an assessment group helped to guard against this possibility. The trial had relatively high dropout rates at 12 weeks, which could have influenced the outcomes in unforeseen ways. These dropout rates are similar to other trials testing digital behavioral interventions,^28,29^ as well as trials of young adults recruited from EDs.^9^ Additionally, dropout rates were not different between treatment arms and were handled using best-practices for multiple imputation to minimize biased effect sizes. This study did not include adolescents or newly licensed individuals, in whom rates of seat belt use are poor. Future research should evaluate the effectiveness of the text messaging intervention in this age group. Despite findings trends of effects at 12 weeks, we were not powered to test significant effects at 12 weeks. Durable intervention effects could potentially be bolstered by running the intervention longer than 6 weeks, consistent with behavioral literature that it may take longer for behaviors to reach automoaticity.^30^ Additionally, the trial was conducted during the unprecedented social disruption of the COVID-19 pandemic, which may have affected vehicle use and seat belt behaviors in unknown ways.

## Conclusions

This randomized clinical trial demonstrated the short-term effectiveness of an automated, interactive text message intervention in promoting consistent seat belt use among young adults. If replicated in other prospective studies of at-risk young adults, a program like SAVE could fill a needed gap in supporting young adults to reduce the public health burden related to unrestrained MVCs.

## Notes

Supplement 1.
Trial Protocol and Statistical Analysis Plan

Supplement 2. **eTable 1.** Univariate Associations Between Characteristics and Missing Web-Based Outcomes at 6 and 12 Weeks **eTable 2.** Seat Belt Cognitions at Baseline, 6 Weeks, and 12 Weeks

Supplement 3.
Data Sharing Statement
