# Supplementary material for: Effectiveness of a Text Message Intervention Promoting Seat Belt Use Among Young Adults: A Randomized Clinical Trial
Source: JAMA Netw Open. 2022 Sep 21;5(9):e2231616. doi: 10.1001/jamanetworkopen.2022.31616 (PMC9494210; doi:10.1001/jamanetworkopen.2022.31616)
Supplement: Supplement 1. — Trial Protocol and Statistical Analysis Plan [file jamanetwopen-e2231616-s001.pdf]

Utilizing Text Messaging to Improve Vehicle Safety Among At-Risk Young Adults – 3 Parallel Randomized Controlled Trials

Acronym: **Safe Vehicle Engament** (**SaVE)** Trials

CLINICAL TRIAL PROTOCOL

Version 1.0

Clinical Trials.Gov Identifier: NCTX

Principal investigator:
Brian Suffoletto, MD MS
Assistant Professor of Medicine, University of Pittsburgh
Attending Physician in Emergency Medicine, UPMC Mercy Hospital

Co-Investigators:
Catherine McDonald, Ph.D.
Kit Delgado, MD
University of Pennsylvannia, Department of Emergency Medicine

Contact Address:
Iroquois Building, Suite 400A
3600 Forbes Ave
Pittsburgh, PA 15261

Phone: 412-901-6892
Fax: 617-647-6999
Email: suffbp@upmc.edu

Funded By:
National Highway Traffic Safety Administration (NHTSA)

SYNOPSIS 5
TRIAL OVERVIEW 6
Feasibility 7
Acceptability 7
Effectiveness .... Error! Bookmark not defined.
STEERING COMMITTEE 8
TRIAL SITES 9
1. BACKGROUND AND SIGNIFICANCE 10
1.1 Scope of the Problem 10
1.2 Existing Vehicle Safety Interventions 10
1.4 Existing Interventions Targeting Cognitive and Motivational Factors of Vehicle Safety 10
1.4 Rationale for SMS Behavioral Interventions 11
2 TRIAL DESIGN 12
2.1 Overview 12
2.2 Setting 12
2.3 Inclusion criteria 12
2.4 Participant Identification 12
2.5. Consent procedures 13
2.6 Contact Information 13
2.7 Baseline Questionnaire 13
2.8 Allocation to Cohorts .... Error! Bookmark not defined.
2.9 SMS & App Onboarding 13
2.10 SMS Run-in 14
2.12 SMS Interventions 14
2.14 Outcome Assessments & Blinding 14
2.15 Compensation 14
2.16 Regulatory Issues 15
2.17 Contingencies and Participant Withdrawal 15
2.18 Study flow diagram 15
3. OUTCOMES 15

3.1 Definitions 15
3.1.1 Primary Outcome 15
3.1.2 Secondary Outcomes: Feasibility 15
3.1.3 Secondary Outcomes: Acceptability 15
3.1.4 Secondary Outcomes: Effectiveness 15
3.2 Rationale for Primary Outcomes 16
3.3 Safety 16
3.3.1 Overview 16
3.3.2 Specific adverse event data collection 16
3.3.3 Adverse Event Reporting 17
4. SAMPLE SIZE CALCULATION AND STATISTICAL ANALYSIS PLAN 17
4.1 Sample size calculation 17
4.2 Statistical analysis plan 17
4.2.1 General considerations 17
4.2.2 Primary Analyses 17
4.2.4 Subgroup analyses 18
6.2.6 Statistical stopping criteria 18
5. DATA COLLECTION AND MANAGEMENT 18
5.1 Data collection process 18
5.2 Variables 18
5.3 Data quality and validity 18
5.4 Data storage and security 18
6.1 RISKS TO HUMAN SUBJECTS 19
7. MONITORING 20
7.1 Institutional Review Board (IRB) 20
7.2 Data Safety and Monitoring 20
8. TIMELINE AND ENROLLMENT 21
8.1 Timeline 21
8.2 Screening & Enrollment 21
9. FUNDING 22
10. PUBLICATIONS 22
11. DATA SHARING 22
12. INTELLECTUAL PROPERTY 22
13. TASKS AND RESPONSIBILITIES 22

Appendices .... Error! Bookmark not defined.
Appendix 1: Abbreviations .... Error! Bookmark not defined.
Appendix 2: Screening Questions 23
Appendix 3: Baseline Questions 25
Appendix 4: SMS Run-In 28

SYNOPSIS

The “Safe Vehicle Engagement (SaVE)” trials are 3 parallel randomized clinical trials that aim to determine the impact of text messaging (SMS) vehicle safety interventions vs. weekly SMS vehicle safety self-monitoring alone on seat belt use, distracted driving and drink driving among young adults identified in the emergency department with risky vehicle behaviors.

TRIAL OVERVIEW

**Title**	Utilizing Text Messaging to Improve Vehicle Safety Behavior Among At-Risk Young Adults – 3 Parallel Randomized Controlled Trials (The SaVE Trials)
**Clinical Trials Number**	NCT
**Sources of monetary or material support**	NHTSA
**Study Sites**	2-sites in the United States (Pittsburgh & Philadelphia)
**Conditions studied**	Seat belt use, distracted driving, drink driving
**Interventions**	Text messaging (SMS) intervention: vehicle safety self-monitoring with performance feedback and goal support
**Comparator**	SMS vehicle safety self-monitoring
**Inclusion criteria**	Young adult (age 18-25 years)<br>• Cohort 1: Any vehicle trip in past 2 weeks where individual reports not using a seat belt<br>• Cohort 2: Any vehicle trip in past 2 weeks where individual reports phone was used to type while driving and car was moving<br>• Cohort 3: Any vehicle trip in past 2 weeks where individual reports driving a vehicle within 3 hours after consuming 3 or more drinks
**Exclusion criteria**	• Member of a protected population (prisoner)<br>• Unable to provide informed consent<br>• No plan to drive and/or ride in a vehicle in the next month<br>• Non-English speaking<br>• No personal mobile phone or planning on changing phone in next 3 months
**Study type**	Interventional<br>Assessment-only run-in (2-week)<br>Allocation to trial cohort: Computerized algorithm to preferentially allocate to seat beltcohort<br>Allocation to treatment: Randomized (1:1)<br>Intervention model: Parallel group<br>Masking: Assessor-blinded
**Target sample size**	500 Participants<br>• Cohort 1: N=300<br>• Cohort 2: N=100<br>• Cohort 3: N=100
**Primary outcome (week 8)**	a. Cohort 1: Any vehicle trip in past 2 weeks where individual reports not using a seat belt<br>b. Cohort 2: Any vehicle trip in past 2 weeks where individual reports phone was used to type while driving and car was moving<br>c. Cohort 3: Any vehicle trip in past 2 weeks where

	individual reports driving a vehicle within 3 hours after consuming 3 or more drinks
**Key secondary outcomes**	*Effectiveness*<br>-Primary outcomes at week 14 (durability)<br>*Feasibility*<br>-Percentage of ED patients who screen positive and agree to enroll.<br>-Percentage of ED patients who enroll who meet run-in criteria.<br>*Acceptability*<br>-Percentage of ED patients enrolled in the Intervention arm who complete at least 50% of the SMS assessments during the intervention period.<br>-Percentage of ED patients enrolled in the Intervention arm who found the program helpful.

STEERING COMMITTEE

Brian Suffoletto, M.D.
Assistant Professor of Emergency Medicine
University of Pittsburgh School of Medicine

Catherine McDonald, Ph.D.
Assistant Professor of Nursing
University of Pennsylvania School of Medicine

Kit Delgado, M.D.
Assistant Professor of Emergency Medicine
University of Pennsylvania School of Medicine

Jean Shope , Ph.D.
Professor
University of Michigan

Jana Nelson
American College of Emergency Physicians
Emergency Medicine Foundation

Cynthia Singh
Director of Grant Development
American College of Emergency Physicians
Emergency Medicine Foundation

Conflicts of interest

The members of the steering committee have no financial conflicts of interest related to the current trial.

TRIAL SITES

<u>Coordinating Center</u>

University of Pittsburgh
Department of Emergency Medicine
Site Investigator: Brian Suffoletto, MD

<u>Enrolling Sites*</u>

Hospital Name	Location	Site Principal Investigator
UPMC Mercy Hospital	Pittsburgh, PA	Brian Suffoletto, MD
UPMC Passavant Hospital	McCandless, PA	Brian Suffoletto, MD
Hospital of the University of Penn	Philadelphia, PA	Catherine McDonald, PhD
Penn Presbyerian Hospital	Philadelphia, PA	Catherine McDonald, PhD

*Depending on study progress, sites may be added or removed in the future.

1. BACKGROUND AND SIGNIFICANCE

1.1 Scope of the Problem

There were around 25,0000 people killed inside vehicles on U.S. roadways during 2016 (NHTSA, 2016) and over 2 million injured in motor vehicle crashes (MVC) (CDC, 2016). A few key behaviors contribute to increased risk of either MVCs or MVC-related injuries: lack of seat belt use, impaired driving (due to drugs and/or alcohol), and distracted driving (due to either other passengers or electronic devices). Among the deaths from MVCs in 2016, 42% were unrestrained passengers (NHTSA, 2016). These behaviors are especially prevalent in young adults. Over 35% of lifetime medical costs for crash injuries (US$6.5 billion of US$18 billion) are attributed to young drivers aged 15 to 29 (CDC, 2014).

1.2 Existing Vehicle Safety Interventions

To date, most primary prevention interventions for young adult vehicle safety have been through multimedia campaigns (Whittam KP et al., 2006), driver training programmes (Lenne LG et al., 2011; Unni P et al., 2017), or public policies such as surveillance with fines for speeding and seat beltuse (Wilson C et al., 2011). Secondary prevention approaches include mandated interventions for offenders such as alcohol Ignition Interlocks (McGinty EE et al., 2017). Recently, improved knowledge of the behavioral drivers of vehicle safety allow for updated interventions incorporating psychological theories and behavior change techniques (Fernandez et al., 2010).

1.3 Behavioral Drivers of Vehicle Safety

Two psychological theories useful in understanding vehicle safety behaviors are the Theory of Planned Behaviour (TPB) and the Health Belief Model (HBM). In the TPB, intentions are influenced by a person's attitude, perceived norms and self-efficacy. The TPB has been shown to explain up to 53% of variance in intention to speed and 40% of variance in speeding behavior (Stead M et al., 2005). In the HBM,the perceived risks/threats of personal injury and the perceived benefits of performing a safety behavior influence the likelihood of performing them. These HBM factors have been shown to be important in sealt belt use (Chaudhary, Solomon, & Cosgrove, 2004; Helweg-Larsen & Sheppard, 2001).

1.4 Existing Interventions Targeting Cognitive and Motivational Factors of Vehicle Safety

Among the few studies have been published testing behavioral interventions for vehicle safety, several have shown that these cognitive and motivational factors of vehicle safety behaviors can be potentially modified. One study showed that an intervention targeting TPB factors resulted in small immediate improvements in vehicle safety (Poulter DR et al., 2010) but effects were not durable over time. Another study by McDonald et al (2018) reported on the development of a digital intervention targeting TPB factors, finding feasible and initial evidence of effects.

1.5 Rationale for SMS Behavioral Interventions

In the US, mobile phones are near ubiquitous, and by the year 2020, 70% of the world's population will use a smartphone (Ericsson, 2015). Text messaging (SMS) is a commonly used digital communication modality that has particular usefulness in reaching individuals to support behavior change (Suffoletto et al.,2017). SMS interventions are ideally suited to deliver microinterventions–interventions that can be completed in a few moments, typically as a repeated administration, tailored to some immediate assessed need or trigger. These microinterventions are more closely in line with the expectations of consumers of digital information, who may routinely interact with fast-paced, user-driven, interactive content. Systematic reviews have found SMS interventions to be effective in short-term behavioral outcomes (Fjedsoe et al., 2009) and supporting preventive health for adolescents specifically (Badway & Kuns, 2017). Our group has shown that SMS interventions incorporating behavior change strategies of self-monitoring with performance feedback and goal support produces reductions in alcohol use in at-risk young adults (Suffoletto et al., 2017).

1.6 SMS Interventions to Reduce Risky Vehicle Behaviors

In this proposal, we plan to design and test three unique SMS interventions each targeting a different vehicle risk behaviors (seat belt use, distracted driving, and impaired driving). They will all use psychological theory and state of the art human-interaction design to optimize engagement and effects. Specifically, the SMS interventions will incorporate periodic (weekly) check-ins to promote self-monitoring of recent risks, goal commitment prompts tailored to past performance to maximize willingness and support gradual behavioral shaping, and performance feedback. Feedback and features will focus on modifying cognitive and motivational factors found in the TPB amnd HBM.

2. TRIAL DESIGN

2.1 Overview

We propose to conduct 3 related randomized, controlled, parallel group, assessor-blind, superiority trials of 6-week text message interventions vs. SMS vehicle safety self-monitoring in young adult participants with risky vehicle behaviors. A total of 500 adult participants will be enrolled: 300 into Cohort 1 (seat belt); 100 into Cohort 2 (distracted driving); 100 into Cohort 3 (drink driving). Each SMS intervention will be desgined to target a single risk behavior. The study is powered to show a difference of 15% in the percentage of subjects reporting seat belt use at week 8. Other cohort trials (i.e. distracted driving, drink driving) and outcomes will be exploratory.

2.2 Setting

The trial will be conducted at 4 hospital emergency departments in Pennsylvania. Additional sites might be recruited if needed.

2.3 Inclusion criteria

Inclusion criteria:

1) Adult participant (age ≥ 18 years & ≤25 years)
    a. <u>Cohort 1</u>: Any vehicle trip in past 2 weeks where individual reports not using a seat belt
    b. <u>Cohort 2</u>: Any vehicle trip in past 2 weeks where individual reports phone was used to type while driving and car was moving
    c. <u>Cohort 3</u>: Any vehicle trip in past 2 weeks where individual reports driving a vehicle within 3 hours after consuming 3 or more drinks

Exclusion criteria:

1) Member of a protected population (prisoner)
2) Unable to provide informed consent
3) No plan to drive and/or ride in a vehicle in the next month
4) Non-English speaking
5) No personal mobile phone or planning on changing phone in next 3 months

<u>Justification of Inclusion and Exclusion Criteria:</u> The inclusion criteria were chosen to isolate three different vehicle risk behaviors in a population with high risk of injuries due to these risks. We chose to be more inclusive than restrictive in these criteria to ensure me meet enrollment goals in the allotted time of funding. The exclusion criteria were chosen to minimize the risk of potential harm to vulnerable populations.

2.4 Participant Identification

In the ED a research associate (RA) will conduct confidential screening of ED patients during breaks in patient medical care. The RA will review ED admissions data form the EMR to identify potential subjects, identifying adult patients 18-25 years of age who receive medical care in the ED. The RA will then ask a clinician (physicians, nurses, or physician extenders) caring for a potential participant to ask the patient if they are interested in speaking with a researcher about a research study. ED clinicians are instructed to refer only patients who are able to provide informed consent (i.e., oriented, able to concentrate, not intoxicated and can understand/remember requirements of the study). We ask clinicians to document in their medical record about the patient’s assent to talk with the RA.

The RA will then discuss the screening and study details with these patients who agree to be approached. Those who wish to see if they are eligible will be asked to complete limited demographics (age, sex, race, and current education), a 13-question screening test to determine presence of vehicle risks using a secure password-protected website. The demographics will be

used to determine how those who screen and/or enroll in our study differ from the general ED population of young adults. *See Appendix for Screen Questions*.

All participants who are screen positive will be provided an information sheet on vehicle risk reduction. *See Appendix for draft of Discharge Instructions*. We will request a waiver of informed consent to view the electronic medical record to identify potential participants by age and a waiver of written consent to ask limited screening questions. Detailed screening logs, with reason(s) for exclusion will be stored in a password-protected database.

2.5. Consent procedures

After it is determined that they meet all inclusion criteria and no exclusion criteria, the participant will be approached for written informed consent by a RA. The RA will provide the participant/representative information regarding the background and significance of the study, eligibility criteria, and a description of the protocol. The consent process may need to be modified based on site-specific IRB recommendations. The name of the study investigator obtaining consent will be clearly documented, and this person will sign the informed consent document and provide the date and time of their signature. Signed copies of the consent form will be given to the participant/surrogate, and the original consent document will be stored in the secure study file. In obtaining and documenting informed consent, each investigator will comply with the applicable regulatory requirements and adhere to the ethical and Good Clinical Practice principles that have their origin in the Declaration of Helsinki.

2.6 Contact Information

Participants will be asked to provide us with their contact information (name, phone number, email). We will also ask them to provide a social security number, as it is required for participant payment.

2.7 Allocation to Cohorts

We will use the risks reported in the screening survey to allocate the participant to their cohort. The overall goal is to meet recruitment goals for all three cohorts simultaneously, but to preferentially allocate individuals to the seat belt group (Cohort 1). Individuals who report only one risk behavior will be allocated to that study cohort. Individuals who report more than one risk factor will be allocated probabilistically. Individuals who screen positive for seat belt risk + one other risk will have a 75% chance of being allocated to Cohort 1. Individuals who screen positive for seat belt risk + two other risks will be allocate as 60% Cohort 1, 20% Cohort 2, 20% Cohort 3. Individuals who screen positive for both other (non-seat belt) risks will be allocated in a 1:1 ratio.

2.8 Baseline Assessment

We will ask participants to complete a questionnaire collecting detailed demographics, vehicle-related risks, and impulsivity traits. This survey takes about 5 minutes to complete *See Appendix for Survey Questions*. We will also ask participants to complete the 5-Trial Adjusting Delay Discounting Task (Kaffaurnus & Bickel, 2014) to assess monetary impulsivity trait. All cohorts will complete identical baseline assessments.

2.9 SMS Onboarding

We will then ask participants to text in a keyword to our program phone number. Only phone numbers that match the phone numbers entered by the RA in the enrollment process wil receive texts. Once this match is recognized, participants will receive several texts welcoming them to the study and describing the 2-week run-in. Each chohort will receive similar yet unique welcome messages tailored to their risk behavior.

2.10 SMS Run-in

All participants will complete a 2-week run-in period where they complete weekly SMS assessments related to their target risk behavior without receiving any feedback or goal support. The primary purpose of the run-in is to exclude noncompliant subjects. Only participants who respond to at least 50% of the SMS queries in week 1 & 2 will be eligible to continue in the study. The secondary purpose is to identify the post-enrollment/ pre-intervention vehicle risk behaviors that may occur as a result of assessment reactivity. We expect that simply the act of enrolling in a study about vehicle risk and completing weekly SMS assessments of risk may result in reduced risk behaviors.

2.11 Allocation to Treatment

Participants in each cohort who meet run-in criteria will be randomized in a 1:1 ratio to either the SMS intervention or SMS self-monitoring in blocks of 4. The randomization will be stratified according to site (i.e. ED). An independent statistician will create the randomization list using a random number generator. The randomization list will be stored in an electronic database to be unblinded once trial enrollment and follow-up periods have been completed.

2.12 SMS Interventions

All three SMS interventions work iteratively primarily through once-weekly dialogue sessions or micro-interventions and use behavior change techniques (BCTs) including self-monitoring with performance feedback and goal support. Each SMS intervention will differ in the content of queries and feedback, which is tailored to the target risk behavior. SMS queries and subject responses are stored and time-stamped in a database. Branching logic is used to tailor SMS feedback and queries. At the completion of 6-weeks, all SMS intervention participants will be asked to complete another 6 weeks of SMS assessments without receiving any feedback or goal support. This is to serve as a washout period so that durability can be assessed at week 14. All queries, feedback libraries and branching logic will be written prior to trial enrollment. *See Appendix for SMS Intervention Material*.

2.13 SMS vehicle safety self-monitoring (i.e. assessment control)

Participants allocated to the control arm will simply continue to receive weekly SMS assessments related to their target risk behavior without receiving any feedback or goal support for 12 more weeks.

2.14 Outcome Assessments & Blinding

The primary outcomes will be the percentage of participants in each treatment arm who report vehicle risks at week 8 (immediate effects) and week 14 (durable effects). Questions are the same as those used in screening. Participants will receive a text prompting them to log in to the secure web site to complete the 21-question survey which should take them no more than 10 minutes to complete. Research associates and investigators will be blinded to the allocation until outcome evaluation. It is not possible to blind subjects given the nature of the intervention. We do not expect any scenarios where emergency unblinding will be necessary.

2.15 Compensation

Participants will be compensated $15 for completing enrollment procedures. We will add $15 if they meet run-in criteria at the end of week 2 and complete the 8-week follow-up assessment. We will add a final $15 if they complete the 14-week assessment.

2.16 Regulatory Issues

We will submit an IRB protocol both for the trial and coordinating center at the University of Pittsburgh. We will also submit an IRB protocol at the Univeristy of Pennsylvania. We expect the trial to be minimial risk to participants, given that the only possible risk will be from potential breach of confidentiality. We will minimize these risks through various strategies.
The trial will be registerted on ClinicalTrials.gov.

2.17 Contingencies and Participant Withdrawal

- In the unlikely event that a participant is discharged from the ED prior to completing enrollment procedures, we will make every attempt to assist the participant in completing them in the following 48 hours. Those participants who still do not complete in this time period will be withdrawn from the study.
- If a participant withdraws from the study, further communication will be stopped. Data collected prior to withdrawal will be maintained but additional data will not be collected.

2.18 Study flow diagram

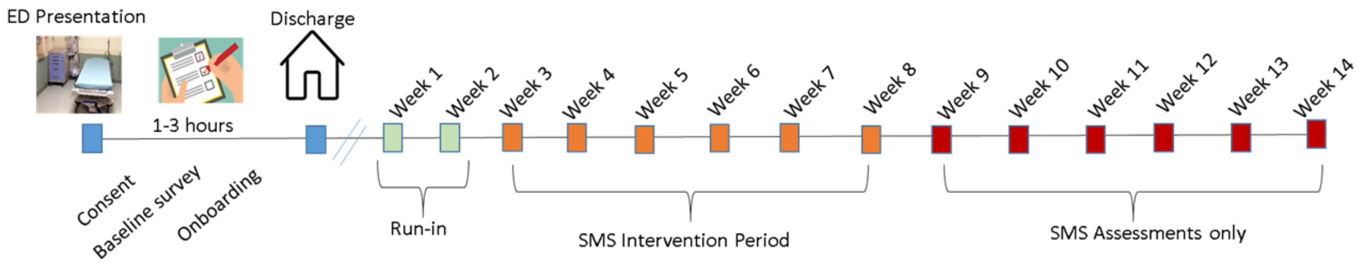

3. OUTCOMES

3.1 Definitions

3.1.1 Primary Outcome

-<u>Percentage of participants who report a vehicle risk at week 8.</u>

d. <u>Cohort 1</u>: Any vehicle trip in past 2 weeks where individual reports not using a seat belt
e. <u>Cohort 2</u>: Any vehicle trip in past 2 weeks where individual reports phone was used to type while driving and car was moving
f. <u>Cohort 3</u>: Any vehicle trip in past 2 weeks where individual reports driving a vehicle within 3 hours after consuming 3 or more drinks

3.1.2 Secondary Outcomes: Feasibility

-Percentage of ED patients who screen positive and agree to enroll
-Percentage of ED patients who enroll who meet run-in criteria.

3.1.3 Secondary Outcomes: Acceptability

-Percentage of ED patients enrolled in the Intervention arm who complete at least 50% of the SMS assessments during the intervention period.
-Percentage of ED patients enrolled in the Intervention arm who found the program helpful.

3.1.4 Secondary Outcomes: Effectiveness

<u>-Percentage with Vehicle Risks at Week 14</u> – We are interested to see if there is any signal of effectiveness once the intervention is "turned-off". This measure of durability is especially critical to understand potential public health benefits.

-Slopes of change over time – We will also examine the average slopes of change over time, specifically examining the slopes of change in the pre-enrollment, run-in (2-weeks), active intervention (6-weeks), and post-intervention (6-weeks) periods.

3.2 Rationale for Primary Outcomes

3.2.1 Overview

We chose to measure self-reported outcomes because objective data on vehicle safety behaviors is difficult to collect, costly, and obtrusive. For example, seat belt use would require in-vehicle equipment or camera monitoring. Alcohol use would require an individual to provide semi-continuous breath alcohol measurements. We chose to assess outcomes over a 2-week timeframe to minimize recall biases associated with longer recall periods but to allow an adequate sampling period where an individual had enough vehicle trips to be considered representative of behaviors. *For list of outcome assessment questions, see Appendix*.

3.2.2 Cohort 1: Any vehicle trip in past 2 weeks where individual reports not using a seat belt. Not wearing a seat belt is associated with higher odds of death or serious injury related to a motor vehicle crash. The SMS intervention aims to encourage consistent use of a seat belt. We will explore whether differences exist between front and rear-seat passenger trips or driver versus passenger trips.

3.2.3 Cohort 2: Any vehicle trip in past 2 weeks where individual reports phone was used to type while driving and car was moving. Typing while driving (TWD) has been shown to increase collision or near-collision event risk by two-fold (Fitch et al., 2013), and studies among younger drivers have found that TWD is associated with 35% slower reaction times (Reed and Robbins, 2008) and a four-fold increase in time spent looking away from the road compared to those driving undistracted (Hosking et al., 2006). We chose to asses not just typing text messages, but any typing on a phone, as there are a number of platforms for communication outside of texting that are commonly used (e.g. Google searches, social media messaging, location-based services).

3.2.4 *Cohort 3*: Any vehicle trip in past 2 weeks where individual reports driving a vehicle within 3 hours of consuming 3 or more drinks. The risk of a motor vehicle crash increases exponentially with elevated blood alcohol content (NHTSA). We chose as an outcome driving a vehicle within 3 hours of consuming 3 or more drinks as this would indicate a high likelihood of a peak BAC of at least 0.05 mg/dl, which would inidicate some psychomotor impairment and elevated risk taking (Van Dyke & Filmore, 2017).

3.3 Safety

3.3.1 Overview

We do not expect, based on the nature of the SMS intervention, to have any serious adverse events. The only forseeable rare adverse event would be to cause a vehicle crash by texting with someone when they are driving. We will minimize this risk by informing all participants to refrain from texting us while driving.

3.3.2 Specific adverse event data collection

To assess specific amd potentially serious adverse events that may be related to the interventions, we will collect data on the following:

3.3.3 Adverse Event Reporting

Any unexpected adverse events will be recorded and reported directly to the appropriate IRB shortly following the event per local protocol.

4. SAMPLE SIZE CALCULATION AND STATISTICAL ANALYSIS PLAN

4.1 Sample size calculation

The study has been powered to have at least 80% power for the primary outcome for Cohort 1 (seat belt use). Based on Sommers et al., 2013, we can likely expect the intervention to increase the percentage of subjects reporting seat belt use by15% on top of self-monioting alone. Using a 2-sample test of proportions, estimating 25% of the intervention participants and 10% of the control (SMS self-monitoring) report always seat beltuse, we would need around 100 subjects per group to show a significant difference between arms. Expecting 10% drop-out in the SMS 2-week run-in and 80% completing 8-week assessments, 300 enrolled in the ED should provide an analyzed sample of 216 (108 per arm).

4.2 Statistical analysis plan

4.2.1 General considerations

The statistical analyses and reporting will adhere to the CONSORT guidelines. All tests will be two-sided, a p-value < 0.05 will be considered significant, and all confidence intervals will have 95% coverage. All analyses will be conducted on a modified intention-to-treat basis only including participants responding to at least one week of SMS assessments during the intervention period. The two groups will be compared in relation to baseline characteristics using descriptive statistics. The persons conducting the statistical analysis will be blinded to the randomized allocation. Groups will be designated as “A” and “B” until all pre-specified analyses are performed and shared with all authors.

4.2.2 Primary Analysis

At baseline, all participants (based on screening criteria) all had the risk factor. At subsequent time points, participants are analyzed as having/not having the same risk factor by randomized group. For our primary analysis, we will examine the proportion of participants in each group (SMS intervention; assessment control) who report the risk behavior using a chi-squared test of proportions. We will then perform a logistical regression, entering any baseline attribute that has univariate association with the outcome in the final model. We will report the difference in percentage with primary outcomes as well as odds ratios (from logistical regressions) with 95% confidence intervals The α level is set at .05 for all statistical tests.

4.2.3 Secondary Analyses

The percentage of ED patients who screen positive and agree to enroll, the percentage of ED patients who enroll who meet run-in criteria, the percentage of ED patients enrolled in the Intervention arm who complete at least 50% of the SMS assessments during the intervention period, and the percentage of ED patients enrolled in the Intervention arm who found the program helpful will be calculated and presented with 95% confidence intervals. We will explore whether individual factors (e.g. sex, race, education) are associated with enrolling, meeting run-in criteria, completing SMS assessments or finding the program helpful using multi-level modeling. We will examine whether perceived norms, perceived control, or perceived risk differed between treatment arms. We will explore the slope of change over time in SMS risk reports using generized estimating equations (GEE) with log link function, examining time, treatment and time*treatment effects. Any individual factors signifiantly associated with SMS assessment completion will be included as

covariates.GEE is the recommended technique to handle non-parametric residuals and missingness associated with repeated-measured data. We will use robust estimates and account for clustering of outcome data within individuals. Results will be presented as odds ratios with 95% confidence intervals.

Handling Missing Outcome Data: If a participant is missing outcome assessment time points (week 8 or week 14), vehicle risk behavior will be imputed based on a pre-defined plan as follows. If a participant has reported that risk via SMS weekly reports, we will use those values for missing weeks. If either of the prior 2 weeks is missing on both web-based recall report or SMS reports, we will impute the missing week based on the worst performance recorded in the weeks either before or after the missing one using SMS values. We believe that this will provide the most conservative estimate of the missing value. Sensitivity analyses will be performed using various other imputation techniques, including multiple chained imputations using baseleine and prior SMS reports.

4.2.4 Subgroup analyses

The analysis will include three pre-defined subgroup analyses for the primary and key secondary outcomes according to 1) participants with high baseline impulsivity (based on delay discount task or self-report (S-UPPS); 2) participants with low perceived danger (defined as those who report No danger associated with target vehicle risk); and 3) participants with low perceived control (i.e. disagree with being able to control behavior) . These cut-off were chosen to represent a population with the highest predicted likelihood of injury due to vehicle risks. The trial is not powered to detect subgroup differences and these will be considered exploratory and hypothesis generating.

4.2.5 Statistical stopping criteria

There will be no formal stopping criteria for efficacy. There will be no predefined stopping criteria for futility since enrollment of the full cohort might allow for detection of efficacy in subgroups or in other outcomes even if the primary outcome is negative.

5. DATA COLLECTION AND MANAGEMENT

5.1 Data collection process

Data collection will be the responsibility of the individual site investigators with oversite from the trial coordinating center. All baseline and outcome variables (i.e. demographics, vehicle risk behaviors) will be obtained prospectively from electronic surveys. Data will be entered directly into the online database software (see below).

5.2 Variables

Measure library is included in the Appendix.

5.3 Data quality and validity

Data quality and validity will be optimized by using a detailed data dictionary which will be distributed to all sites. Data quality will be monitored both centrally by the coordinating site and locally by each site principal investigator.

5.4 Data storage and security

All data will be stored and secured by Jack Doman, the Director of the Office of Academic Computing (OAC) at Western Psychiatric Institute and Clinic (WPIC). Baseline data will be entered into *Web Data Xpress*, a web-based data entry system that enables users to add and edit records in a database. By using Secure Sockets Layer (SSL) and user authentication, users can be assured that data transmitted over the internet is safe and secure. The system to send, receive and process text messages, was developed on the OAC SQL Server 2005 system with permissions granted to specific EM faculty and staff to access the data for a given project through a Microsoft

Access front-end. Through these mechanisms, as well as relevant training for all involved parties, participant confidentiality will be safeguarded.

The consent form and other trial documents for each participant will initially be stored in a secure, locked place at the individual sites. Participating sites will be responsible for maintaining their own trial documents and study materials. Trial documents generated at the Coordinating Center will be maintained the Coordinating Center. Following completion of the trial, documents will be maintained for a period of at least 7-years at each site (or longer depending on local IRB guidelines).

6.1 RISKS TO HUMAN SUBJECTS

Information obtained at the time of screening and assessment may pose psychological risks and some of the questions may be considered sensitive in nature and may cause emotional distress. We will be collecting information that if disclosed could have adverse consequences for subjects or damage their financial standing, employability, insurability, or reputation. Additionally, we are enrolling underage individuals, which risk disclosure of illegal activity associated with alcohol and/or drug use. Participants face some risk of loss of confidentiality due to the nature of data acquisition through the internet and cell phone SMS. First, participants may have information on their mobile phone that is seen by someone else. As well, there may be the rare risk that someone not associated with this research sees their medical record or data as a result of their participation in this research. Overall, the likelihood of any of these risks causing a subject any serious discomfort or inconvenience is very low. Confidentiality of information will be maximized in accordance with HIPPA regulations.

6.2 ADEQUANCY OF PROTECTION AGAINST RISK

6.2.1 Recruitment and Informed Consent: Eligible patients will be identified by a Research Associate with clinical appointments at enrolling hospitals. The RA will conduct confidential screening of ED patients during breaks in patient medical care. All responses to screening questions will be recorded on a secure web site. The Primary Investigator will audit screenings and will be available to the RA during screening. The RA will approach all permission-granting, potentially eligible patients to obtain informed consent for study *screening*. To ensure privacy, family or other individuals accompanying the patient will be asked to leave the area prior to the screening procedures and patients will be reminded that other people may be able to overhear the conversation. The RA will provide tablet computer for participants to complete screening questionnaire immediately after informed consent. Screening will include review of inclusion/exclusion criteria and basic demographic information to determine how those who screen positive differ from those who screen negative. Interested and eligible individuals will be asked to complete written informed consent. The computer shows the RA whether the participant is eligible or not, and whether the risks were present or not. The RA will then read a standard script based on eligibility and offer eligible patients study participation, obtaining written informed consent. The RA will present an oral and written description of the study as part of informed consent, including a description of the project procedures, potential risks and benefits, and confidentiality. The RA will review any questions the patient may have during the consent process. Patients will be informed that they will be compensated for their time during the research assessments.

6.2.2 Protection Against Risk: In order to protect study participants form potential risks related to the loss of confidentiality and due to any discomfort that they may experience in answering any questions, the following steps will be taken: 1) Patients will be told that they can withdraw from the study at any time by contacting us. 2) All information provided by the patient will be referenced to a subject ID# and will be kept in locked file cabinets. The patient’s ID# can be connected to the subject’s name only through a single master file, accessible only by the Primary Investigator. All data and files will be entered on computers protected by passwords and stored in a locked office. 3) All research staff will be trained in the importance of maintaining confidentiality and will undergo annual mandatory training of human subjects research. 4) All patient data will be presented in aggregate and no individuals will be identified individually. Additionally, we will make every effort to ensure that subject information is protected so only

authorized persons can see their information. All messages sent from subjects to our phone number will be catalogued and encrypted, stored per UPMC security standards. All participants will be advised to set up password protection on their cell phones and to erase messages after responding to minimize the chance of loss of private information. We state explicitly in the consent that we will not be able to respond in real-time to any concern a patient has that is outside the expected responses that is sent to our phone number, nor any text message outside the scope of the question we ask. Immediate or emergency text-messages that are received and outside the scope of the questions we sent will all receive a standardized text message, stating "We appreciate your participation. If you have an emergency, please call 911." As well, all messages are archived in our database and will be reviewed at least every 2 days by the investigators. At the end of this study, the data key will be destroyed.

6.2.3 Certificte of Confidentiality: Because we are collecting data that is sensitive, we will seek a Certificate of Confidentiality from the NIH.

6.2.4 Potential Benefits to Individual Participants: All participants (and those who screen positive) will be offered an informational sheet on vehicle risks. Particiapnts in both treatment arms will also be prompted to self-monitor their vehicle risks over a period of 14 weeks, which also will likely result in reduced risks thorugh assessment reactivity and awareness. Assuming our hypothesis is correct, individual participants enrolled in our study and randomized to the treatment arm will also benefit in learning improved vehicle safety behaviors.

6.2.4 Potential Benefits to Society: Risky vehicle behaivors are associated with increased probability of serious injury due to motor vehicle crashes. No current existing behavioral support tool exists to reduce these risky behviors. Our study, assuming our hypothesis is confirmed, will provide strong support for the widespread adoption of an SMS program for young adults with these risks. This, in turn, could significantly reduce the global burden of injury-related morbidity and mortality.

7. MONITORING

7.1 Institutional Review Board (IRB)

The study will be reviewed and approved by the IRB at each participating site.

7.2 Data Safety and Monitoring

The proposed study will be monitored to enhance the safety of study participants. Although minimal risks are expected for participants in the study, all data and research enrollment processing will be audited at least monthly by the Departmental Clinical Research Meeting (DCRM) of the Department of Emergency Medicine. This DCRM includes senior researchers in the Department of Emergency Medicine and is responsible for continual review of all non-exempt studies involving patients. At least one investigator of this study will attend these meetings. All members of the DCRM have considerable research and clinical experience to evaluate the study's recruitment and retention procedures, to monitor any potential significant benefits or risks that may occur so as to warrant the early termination of the study and to monitor study progress. Additionally, bi-weekly meetings will be held with primary investigators and research assistants to review research process. Any significant issues or variations from protocol related to this study will be reported to Department of Emergency Medicine DCRM and to the University of Pittsburgh Institutional Review Board in a timely fashion. Upon study renewal, a monitoring report will be provided to the IRB.

8. TIMELINE AND ENROLLMENT

8.1 Timeline

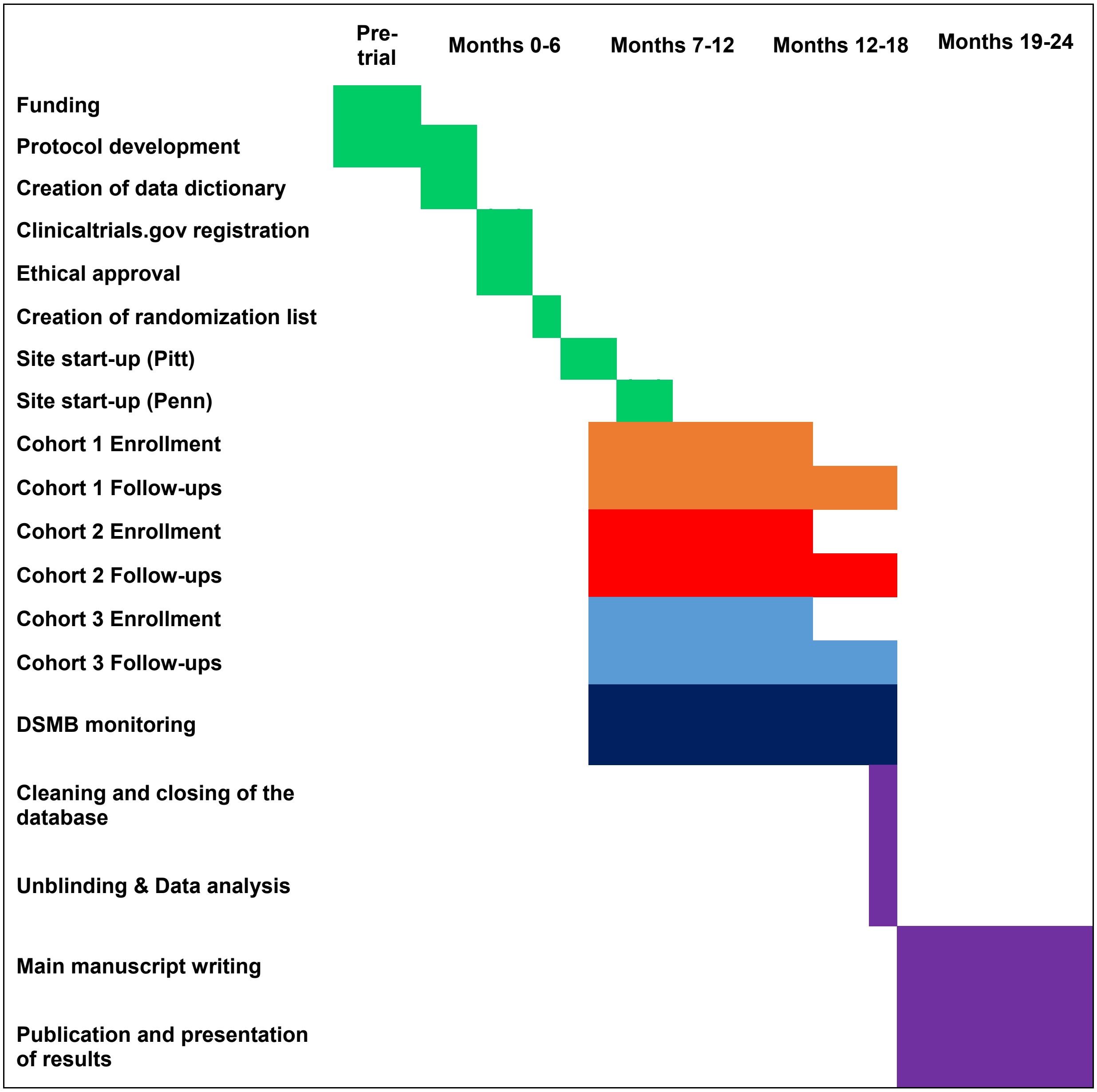

8.2 Screening & Enrollment

Enrollment at each site will be continuously monitored by the site investigator and the principal investigator. Each site will maintain a screening log including all participants who meet all eligibility criteria at that site. A standardized screening log will be kept using WebDataXpress, thus allowing for continuous updating of the screening log and will allow capture of all screening failures.

Enrollment will be competitive (i.e. without specific enrollment caps). Number of enrollments at each site will be shared with all sites on a monthly basis. Sites will be expected to complete all elements of the online screening and enrollment forms for each subject. In the case that a site continuously underperforms despite troubleshooting and feedback, the Investigators will evaluate whether enrollment will continue at that site.

9. FUNDING

Funding for the present trial is provided by NHTSA in partnership with EMF. The funding agencies have no role in the design and conduct of the study, collection, management, analysis, and interpretation of the data, preparation, review, or approval of the manuscript, or the decision to submit the manuscript for publication.

10. PUBLICATIONS

We plan to write 3 separate main manuscripts describing findings related to Cogorts 1,2, &3. The manuscripts will adhere to the CONSORT guidelines. The principal investigator will be responsible for assigning authorship position and will follow authorship guidelines from the International Committee of Medical Journal Editors. At a minimum, all members of the Investigator Team will be included in the primary author list. The main results will be presented at an international conference. The trial results will be shared with participating sites and via press releases but not directly with the participants.

11. DATA SHARING

Six months after the publication of the last results, all de-identified individual participant data will be made available for data sharing. Procedures, including re-coding of key variables, will be put in place to allow for complete de-identification of the data. All relevant trial-related documents, including the protocol, data dictionary, and the main statistical code, will be shared along with the data. There will be no predetermined end date for the data sharing. Data will be available for any research purpose to all interested parties who have approval from an independent ethics review committee and who have a methodological sound proposal as determined by the steering committee of the current trial. Interested parties will be able to request the data by contacting the principal investigator. Authorship of publications emerging from the shared data will follow standard authorship guidelines from the International Committee of Medical Journal Editors and might or might not include authors from the steering committee depending on the nature of their involvement.

12. INTELLECTUAL PROPERTY

We will submit a disclosure of intellectual proprerty to the University of Pittsburgh between primary analyses and publication submission. Inventors to be listed include Brian Suffoletto, Catherine McDonald and Kit Delgado.

13. TASKS AND RESPONSIBILITIES

Principal investigator: Overall responsibility for protocol development, intervention development, budget overview, data dictionary development, ethical approval, trial registration, daily management, trial oversight and collection of adverse events, and the data and safety monitoring board, assessment of overall recruitments, potential recruitment of additional sites, data analysis, and dissemination and presentation of results.

Co-Investigators: Site-specific enrollment, education of personnel at participating sites, reporting of site-specific issues or challenges to the principal investigator, participant consent for data collection, collecting and reporting data regarding adverse events. Also protocol development, data dictionary development, trial oversight, dissemination of results.

Steering committee: Protocol development, data dictionary development, trial oversight, dissemination of results.

Appendix: Screening Questions

**Question**	**How old are you?**	**18 years old**	**19 years old**	**20 years old**	**21 years old**
Code	age	0	1	2	3
**Question**	**What is your sex?**	**Female**	**Male**		
Code	sex	0	1		
**Question**	**Are you Hispanic or Latino?**	**No**	**Yes**		
Code	hisp	0	1		
**Question**	**What is your race? (Select one or more responses.)**	**Black or African American**	**White**	**Asian**	**Other**
Code	brace, wrace, asian, rother	0/1	0/1	0/1	0/1
**Question**	**Are you currently enrolled in school?**	**No**	**Yes, High School**	**Yes, College**	
Code	school	0	1	2	
	**<u>The following questions ask you to recall times you have been in a car over the past 2 weeks.</u>**				
**Question**	**How often have you driven a car?**	**Never**	**A few times**	**Most days**	**Every day**
Code	driver	0	1	2	3
**Question**	**How often did you wear a seat belt when you drove a car?**	**Never**	**A few times**	**Most of the time**	**Always**
Code	drive_seatbelt	0	1	2	3
**Question**	**How often have you been a passenger in the <u>front seat</u> of a car?**	**Never**	**A few times**	**Most days**	**Every day**
Code	pass_front	0	1	2	3
**Question**	**How often did you wear a seat belt when you were a passenger in the <u>front seat</u>?**	**Never**	**A few times**	**Most of the time**	**Always**
Code	pass_seatbelt1	0	1	2	3
	**How often have you been a passenger in the <u>back seat</u> of a car?**	**Never**	**A few times**	**Most days**	**Every day**
	pass_back	0	1	2	3
	**How often did you wear a seat belt when you were a passenger in the <u>back seat?</u>**	**Never**	**A few times**	**Most of the time**	**Always**
	pass_seatbelt2	0	1	2	3
**Question**	**How often did you type on your phone while you were driving and when the car was moving?**	**Never**	**A few times**	**Most of the time**	**Always**
Code	distract	0	1	2	3
**Question**	**Have you driven a vehicle within 3 hours after consuming 3 or more alcoholic drinks?**	**No**	**Yes**		
Code	drinks	0	1		
	**<u>Thanks! Please hand the iPAD back to the Researcher.</u>**				
	**RA enters code: SAVE**				
	**Program displays which risks+**				
**Script**	**RA tells pt they are potentially eligible for study or thanks them for their time.**				
	**If eligible pt interested, RA asks the following:**				

		No	Yes
**Question**	**Do you have a personal cell phone with text messaging ?**	**No**	**Yes**
Code	sms	0	1
**Question**	**Do you plan to drive and/or ride in a vehicle in the next month?**	**No**	**Yes**
Code	future	0	1
**Question**	**Do you plan to change phone numbers in the next 3 months?**	**No**	**Yes**
Code	phone	0	1

Logic If sms=0 or future=0 or phone=1, they are excluded.

RA either thanks them for their time or starts informed consent.

RA goes to WebDataXpress site

Appendix: Baseline Questions

**Question**	**Who do you live with?**	**I live alone**	**Friend(s), same sex**	**Friend(s), other sex**	**Parents or family**		
Code	livew	0	1	2	3		
**Question**	**Are you currently employed?**	**No**	**Yes, part-time**	**Yes, full time**			
Code	emply	0	1	2			
**Question**	**Check off all the reasons you have not used a seat belt**	**Uncomfortable**	**Forgot**	**Don't think they help**	**Like freedom**	**Other**	**I always wear my seat belt**
Code	seatbelt_reasons	0	1	2	3	4	5
**Question**	**If other, state reasons**						
Code	seatbelt_other						
**Question**	**How often do your friends use their seat belt?**	**Never**	**Rarely**	**Most of the time**	**Always**		
Code		0	1	2	3		
**Question**	**How dangerous is it to not wear a seat belt?**	**Not at all**	**Somewhat**	**Very**	**Completely**		
Code	danger_seatbelt	0	1	2	3		
**Question**	**How much do you agree: I have complete control over whether I wear a seat belt.**	**Strongly Disagree**	**Disagree**	**Somewhat Agree**	**Mostly agree**	**Strongly Agree**	
Code	control_sb	0	1	2	3	4	
**Question**	**Check off all the reasons you have typed on your phone while driving?**	**I could not wait**	**I don't think it affects my driving**	**It was an emergency**	**It is fun**	**I get bored driving**	**I do not use my phone while driving**
Code	twd_reasons	0	1	2	3	4	5
**Question**	**How often do your friends type on their phones while driving?**	**Never**	**Rarely**	**Most of the time**	**Always**		
Code		0	1	2	3		
**Question**	**How dangerous is it to type on your phone while driving?**	**Not at all**	**Somewhat**	**Very**	**Completely**		
Code	danger_twd	0	1	2	3		
**Question**	**How much do you agree: I have complete control over whether I type on my phone while driving.**	**Strongly Disagree**	**Disagree**	**Somewhat Agree**	**Mostly agree**	**Strongly Agree**	
Code	control_twd	0	1	2	3	4	
**Question**	**Check off all the reasons you have driven a car after drinking alcohol?**	**I had no other opiton**	**I don't think it affects my driving**	**It's not a big deal**	**I am careful**	**It is fun**	**I do not drive after dirnking alcohol**
Code	dd_reasons	0	1	2	3	4	5
**Question**	**How often do your friends drive after drinking alcohol?**	**Never**	**Rarely**	**Most of the time**	**Always**		
Code	friends_dd	0	1	2	3		
**Question**	**How dangerous is it to drive soon after drinking more 3 or more alcoholic drinks?**	**Not at all**	**Somewhat**	**Very**	**Completely**		
Code	danger_dd	0	1	2	3		

**Question**	**How much do you agree: I have complete control over whether I type on my phone while driving.**	**Strongly Disagree**	**Disagree**	**Somewhat Agree**	**Mostly agree**	**Strongly Agree**
Code	control_dd	0	1	2	3	4

**Question**	**Answer the following questions about yourself.**	**Strongly Disagree**	**Disagree**	**Somewhat Agree**	**Mostly agree**	**Strongly Agree**
cat code	S-UPPS	0	1	2	3	4
Question	**When I feel bad, I will often do things I later regret in order to make myself feel better now.**					
Question	**Sometimes when I feel bad, I can't seem to stop what I am doing even though it is making me feel worse.**					
Question	**When I am upset I often act without thinking.**					
Question	**When I feel rejected, I will often say things that I later regret.**					
Question	**I generally like to see things through to the end.**					
Question	**Unfinished tasks really bother me.**					
Question	**Once I get going on something I hate to stop.**					
Question	**I finish what I start.**					
Question	**My thinking is usually careful and purposeful.**					
Question	**I like to stop and think things over before I do them.**					
Question	**I tend to value and follow a rational, "sensible" approach to things.**					
Question	**I usually think carefully before doing anything.**					
Question	**I quite enjoy taking risks.**					
Question	**I welcome new and exciting experiences and sensations, even if they are a little frightening and unconventional.**					
Question	**I would like to learn to fly an airplane.**					
Question	**I would enjoy the sensation of skiing very fast down a high mountain slope.**					
Question	**When I am in great mood, I tend to get into situations that could cause me problems.**					
Question	**I tend to lose control when I am in a great mood.**					
Question	**Others are shocked or worried about the things I do when I am feeling very excited.**					
Question	**I tend to act without thinking when I am really excited.**					

Question	**How long have you been driving a car?**	Less than 6 months	6 months to 1 year	1-2 years	2-5 years	More than 5 years	never driven
Code	long_dr	0	1	2	3	4	5
Question	**How many times in the last 12 months have you received a traffic ticket? (Not including parking tickets)**						
Code	tix	#					
Question	**As a driver of a car, have you been in a crash in the past 12 months?**						
Code	crash	#					

Question	**How many days over the last month have you drank more than (3 drinks for women/ 4 drinks for men)?**	
Code	lmbinge	#

Question	**How many days over the last month have you used cannabis (marijuana, pot, has, grass, etc)?**	
Code	Imbinge	#

<u>Thanks, This ends your baseline survey. Please hand me back to the Researcher.</u>

Appendix: SMS Run-In: Seat belt

Day & Time	Incoming	Outgoing
Enrollment		Hi [_NAME_]. Welcome to to the SaVE Study
		For the next 2 weeks, each Sunday, we will ask you about your seat belt use in the past week
		If you complete at least 50% of the text queries, you will be eligible for the rest of the study
	Any response	If at any time you wish to stop receiving texts, just text us "stop"
Sunday, 4pm		Hi [_NAME_], it's theSaVE Team checking in<br>**How often have you been a passenger or driver in a car the past week? 0=never; 1=a few times; 2=most days; 3=every day**
2 hours	[Missing] x1	We missed your response. Text us how often you have been a passenger or driver in a car the past week
2 hours	[Missing] x2	You must be busy. We will check in next week
	[Nonsense]	We don't understand. Please text us a response option: 0=never; 1=a few times; 2=most days; 3=every day
	0	OK
	1,2,3	**How often did you wear a seat belt? 0=never; 1=a few times; 2=most of the time; 3=every time**
1 hour	[Missing] x1	We missed your response. Text us how often you wore a seatbelt
1 hour	[Missing] x2	You must be busy. We will check in next week
	[Nonsense]	We don't understand. Please text us a response option: 0=never; 1=a few times; 2=most of the time; 3=every time
	0,1,2,3	Thanks
Day 15 (Monday)		Due to your low response rate, you will be withdrawn from the SaVE Study
		For further information about seat belt safety, go to www.NHTSA.gov

Appendix: SMS Run-In: Typing While Driving (TWD)

Day & Time	Incoming	Outgoing
Enrollment		Hi [_NAME_]. Welcome to to the SaVE Study
		For the next 2 weeks, each Sunday, we will ask you about your seatbelt use in the past week
		If you complete at least 50% of the text queries, you will be eligible for the rest of the study
	Any response	If at any time you wish to stop receiving texts, just text us "stop"
Sunday, 4pm		Hi [_NAME_], it's theSaVE Team checking in<br>**How often have you driven a car the past week? 0=never; 1=a few times; 2=most days; 3=every day**
2 hours	[Missing] x1	We missed your response. Text us how often you have been a passenger or driver in a car the past week?
2 hours	[Missing] x2	You must be busy. We will check in next week
	[Nonsense]	We don't understand. Please text us a response option: 0=never; 1=a few times; 2=most days; 3=every day
	0	OK.

	1,2,3	**When driving, how often did you type on your phone when the car was moving? 0=never; 1=a few times; 2=most of the time; 3=every time**
1 hour	[Missing] x1	We missed your response. Text us how often you typed on your phone when the car was moving
1 hour	[Missing] x2	You must be busy. We will check in next week
	[Nonsense]	We don't understand. Please text us a response option: 0=never; 1=a few times; 2=most of the time; 3=every time
	0,1,2,3	Thanks
Day 15 (Monday)		Due to your low response rate, you will be withdrawn from the SaVE Study
		For further information about safe vehicle use, go to www.NHTSA.gov

Appendix: SMS Run-In: Drink Driving

Day & Time	Incoming	Outgoing
Enrollment		Hi [_NAME_]. Welcome to to the SaVE Study<br>For the next 2 weeks, each Sunday, we will ask you about your seatbelt use in the past week
		If you complete at least 50% of the text queries, you will be eligible for the rest of the study
	Any response	If at any time you wish to stop receiving texts, just text us "stop"
Sunday, 4pm		Hi [_NAME_], it's theSaVE Team checking in<br>**How often have you driven a car the past week? 0=never; 1=a few times; 2=most days; 3=every day**
2 hours	[Missing] x1	We missed your response. Text us how often you have been a passenger or driver in a car the past week?
2 hours	[Missing] x2	You must be busy. We will check in next week
	[Nonsense]	We don't understand. Please text us a response option: 0=never; 1=a few times; 2=most days; 3=every day
	0	OK
	1,2,3	**Have you driven a vehicle within 3 hours after consuming 3 or more alcoholic drinks?**
1 hour	[Missing] x1	We missed your response. Text us Yes or No.
1 hour	[Missing] x2	You must be busy. We will check in next week
	[Nonsense]	We don't understand. Please text us either Yes or No
	No	Thanks
Day 15 (Monday)		Due to your low response rate, you will be withdrawn from the SaVE Study
		For further information about safe vehcile use, go to www.NHTSA.gov

Appendix: SMS Interventions: Seat belt Safety

Day & Time	Incoming	Outgoing
**Qualified Week 3, Monday, 5pm**		
Qualified		Congratulations [Name]! You have qualified to continue the SaVE Study
		For the next 6 weeks, we will help you set goals and provide you personalized feedback on your seat belt use
		We will check in next Sunday. Until then, take care
**Week 3-8: Sunday, 3pm**		
		Hi [_NAME_], it's the SaVE Study Team checking in<br>**How often have you been a passenger or driver in a car the past week? 0=never; 1=a few times; 2=most days; 3=every day**
2 hours	[Missing] x1	We missed your response. Text us how often you have been a passenger or driver in a car the past week?
2 hours	[Missing] x2	You must be busy. We will check in next week
	[Nonsense]	We don't understand. Please text us a response option: 0=never; 1=a few times; 2=most days; 3=every day
	0	OK
	1,2,3	**When in the car, how often did you wear a seat belt? 0=never; 1=a few times; 2=most of the time; 3=every time**
1 hour	[Missing] x1	We missed your response. Text us how often you wore a seat belt
1 hour	[Missing] x2	You must be busy. We will check in next week
	[Nonsense]	We don't understand. Please text us a response option: 0=never; 1=a few times; 2=most of the time; 3=every time
If Goal Not Set	3	[Posiitve Reinforcement Message]
	0,1,2	[Social Norms Message]
If Goal Set	Goal met	[Goal Met Message]
	Goal not met	[Goal Not Met Message]
		**Would you be willing to commit to a goal to wear a seat belt every time this week?**
1 hour	[Missing] x1	We missed your response. Text us whether you'd be willing to set a goal?
1 hour	[Missing] x2	You must be busy. We will check in next week
	[Nonsense]	We don't understand. Please text us either yes or no
No Goal	No	[No Goal Roll Library Message]
Goal +	Yes	[Goal Reinforcement Message]
**Random 2 days per week, 4pm**		
If goal -		[Seat belt Safety Info Message]
If goal +		[Goal Reminder Message]

Appendix: SMS Interventions: TWD Safety

Day & Time	Incoming	Outgoing
**Qualified Week 3, Monday, 5pm**		
Qualified [At least 1 Sunday Assessment complete]		Congratulations [Name]! You have qualified to continue the SaVE Study
		For the next 6 weeks, we will help you set goals and provide you personalized feedback on your driving safety
		We will check in next Sunday. Until then, take care.
**Week 3-8: Sunday, 3pm**		
		Hi [_NAME_], it's the SaVE Study Team checking in. **How often have you driven a car the past week? 0=never; 1=a few times; 2=most days; 3=every day**
2 hours	[Missing] x1	We missed your response. Text us how often you have been a passenger or driver in a car the past week?
2 hours	[Missing] x2	You must be busy. We will check in tomorrow.
	[Nonsense]	We don't understand. Please text us a response option: 0=never; 1=a few times; 2=most days; 3=every day
	0	OK.
	1,2,3	**When driving, how often did you type on your phone when the car was moving? 0=never; 1=a few times; 2=most of the time; 3=every time**
1 hour	[Missing] x1	We missed your response. Text us how often did you type on your phone while the car was moving
1 hour	[Missing] x2	You must be busy. We will check in tomorrow.
	[Nonsense]	We don't understand. Please text us a response option: 0=never; 1=a few times; 2=most of the time; 3=every time
If Goal Not Set	0	[Posiitve Reinforcement Message]
	1,2,3	[Social Norms Message]
If Goal Set	Goal met	[Goal Met Message]
	Goal not met	[Goal Not Met Message]
		**Would you be willing to commit to a goal to refrain from typing on your phone while driving this week?**
1 hour	[Missing] x1	We missed your response. Text us whether you'd be willing to set a goal?
1 hour	[Missing] x2	You must be busy. We will check in later.
	[Nonsense]	We don't understand. Please text us either yes or no
No Goal	No	[No Goal Roll Library Message]
Goal +	Yes	[Goal Reinforcement Message]
**Random 2 days per week, 4pm**		
If goal -		[Distracted Driving Info Message]
If goal +		[Goal Reminder Message]

Appendix: SMS Interventions: Drink Driving Safety

Incoming	Outgoing
	Congratulations [Name]! You have qualified to continue the SaVE Study
	For the next 6 weeks, we will help you set goals and provide you personalized feedback on your driving safety
	We will check in next Sunday. Until then, take care
	Hi [_NAME_], it's the SaVE Study Team checking in
	**How often have you driven a car the past week? 0=never; 1=a few times; 2=most days; 3=every day**
[Missing] x1	We missed your response. Text us how often you driven a car the past week?
[Missing] x2	You must be busy. We will check in tomorrow
[Nonsense]	We don't understand. Please text us a response option: 0=never; 1=a few times; 2=most days; 3=every day
0	OK
1,2,3	**Have you driven a vehicle within 3 hours after consuming 3 or more alcoholic drinks?**
[Missing] x1	We missed your response. Text us whether you have driven within 3 hours of drinking 3 or more alcoholic beverages.
[Missing] x2	You must be busy. We will check in next week
[Nonsense]	We don't understand. Please text us Yes or No.
No	[Posiitve Reinforcement Message]
Yes	[Social Norms Message]
Goal met	[Goal Met Message]
Goal not met	[Goal Not Met Message]
	**Would you be willing to commit to a goal to not drive after drinking this week?**
[Missing] x1	We missed your response. Text us whether you'd be willing to set a goal?
[Missing] x2	You must be busy. We will check in next week
[Nonsense]	We don't understand. Please text us either yes or no
No	[No Goal Roll Library Message]
Yes	[Goal Reinforcement Message]
	[Drink Driving Info Message]
	[Goal Reminder Message]

Appendix: 8-Week Assessment

	**<u>The following questions ask you to recall times you have been in a car over the past 2 weeks.</u>**							
**Question**	**How often have you driven a car?**	**Never**	**A few times**	**Most days**	**Every day**			
Code	driver	0	1	2	3			
**Question**	**How often did you wear a seat belt when you drove a car?**	**Never**	**A few times**	**Most of the time**	**Always**			
Code	drive_seatbelt	0	1	2	3			
**Question**	**How often have you been a passenger in the front seat of a car?**	**Never**	**A few times**	**Most days**	**Every day**			
Code	pass_front	0	1	2	3			
**Question**	**How often did you wear a seat belt when you were a passenger in the front seat?**	**Never**	**A few times**	**Most of the time**	**Always**			
Code	pass_seatbelt1	0	1	2	3			
**Question**	**How often have you been a passenger in the back seat of a car?**	**Never**	**A few times**	**Most days**	**Every day**			
Code	pass_back	0	1	2	3			
**Question**	**How often did you wear a seat belt when you were a passenger in the back seat?**	**Never**	**A few times**	**Most of the time**	**Always**			
Code	pass_seatbelt2	0	1	2	3			
**Question**	**How often did you type on your phone while you were driving and when the car was moving?**	**Never**	**A few times**	**Most of the time**	**Always**			
Code	distract	0	1	2	3			
**Question**	**What's the most number of alcoholic drinks you've had prior to driving on any occasion?**	**#**						
Code	drinks	#						

**Question**	**Check off all the reasons you have not used a seat belt**	**Uncomfortable**	**Forgot**	**Don't think they help**	**Like freedom**	**Other**	**I always wear my seat belt**	
Code	seatbelt_reasons	0	1	2	3	4	5	
**Question**	**If other, state reasons**							
Code	seatbelt_other							
**Question**	**How often do your friends use their seat belt?**	**Never**	**Rarely**	**Most of the time**	**Always**			
Code		0	1	2	3			
**Question**	**How dangerous is it to not wear a seat belt?**	**Not at all**	**Somewhat**	**Very**	**Completely**			
Code	danger_seatbelt	0	1	2	3			
**Question**	**How much do you agree: I have complete control over whether I wear a seat belt.**	**Strongly Disagree**	**Disagree**	**Somewhat Agree**	**Mostly agree**	**Strongly Agree**		
Code	control_sb	0	1	2	3	4		
**Question**	**Check off all the reasons you have typed on your phone while driving?**	**I could not wait**	**I don't think it affects my driving**	**It was an emergency**	**It is fun**	**I get bored driving**	**I do not use my phone while driving**	**I do not drive**
Code	twd_reasons	0	1	2	3	4	5	6
**Question**	**How often do your friends type on their phones while driving?**	**Never**	**Rarely**	**Most of the time**	**Always**			
Code		0	1	2	3			
**Questi**	**How dangerous is it to type on your phone while driving?**	**Not at all**	**Somew**	**Very**	**Complet**			

**on**			**hat**		**ely**			
Code	danger_twd	0	1	2	3			
**Question**	**How much do you agree: I have complete control over whether I type on my phone while driving.**	**Strongly Disagree**	**Disagree**	**Somewhat Agree**	**Mostly agree**	**Strongly Agree**		
Code	control_twd	0	1	2	3	4		
**Question**	**Check off all the reasons you have driven a car after drinking alcohol?**	**I had no other opiton**	**I don't think it affects my driving**	**It's not a big deal**	**I am careful**	**It is fun**	**I do not drive after dirnking alcohol**	**I do not drive**
Code	dd_reasons	0	1	2	3	4	5	6
**Question**	**How often do your friends drive after drinking alcohol?**	**Never**	**Rarely**	**Most of the time**	**Always**			
Code	friends_dd	0	1	2	3			
**Question**	**How dangerous is it to drive soon after drinking more 3 or more alcoholic drinks?**	**Not at all**	**Somewhat**	**Very**	**Completely**			
Code	danger_dd	0	1	2	3			
**Question**	**How much do you agree: I have complete control over whether I type on my phone while driving.**	**Strongly Disagree**	**Disagree**	**Somewhat Agree**	**Mostly agree**	**Strongly Agree**		
Code	control_dd	0	1	2	3	4		

[redacted]

**Question**	**Did you find the text message program helpful?**	**Not at all**	**Somewhat**	**Very much**
Code	helpful	0	1	2
**Question**	**Would you recommend the program to others?**	**No**	**Yes**	
Code	recom	0	1	
**Question**	**Any other comments you'd like to provide that could help us make the text message program better?**			
Code	comments			

Appendix: 14-Week Assessment

	**<u>The following questions ask you to recall times you have been in a car over the past 2 weeks.</u>**				
**Question**	**How often have you driven a car?**	**Never**	**A few times**	**Most days**	**Every day**
Code	driver	0	1	2	3
**Question**	**How often did you wear a seat belt when you drove a car?**	**Never**	**A few times**	**Most of the time**	**Always**
Code	drive_seatbelt	0	1	2	3
**Question**	**How often have you been a passenger in the <u>front seat</u> of a car?**	**Never**	**A few times**	**Most days**	**Every day**
Code	pass_front	0	1	2	3
**Question**	**How often did you wear a seat belt when you were a passenger in the <u>front seat</u>?**	**Never**	**A few times**	**Most of the time**	**Always**
Code	pass_seatbelt1	0	1	2	3
	**How often have you been a passenger in the <u>back seat</u> of a car?**	**Never**	**A few times**	**Most days**	**Every day**
	pass_back	0	1	2	3
	**How often did you wear a seat belt when you were a passenger in the <u>back seat?</u>**	**Never**	**A few times**	**Most of the time**	**Always**
	pass_seatbelt2	0	1	2	3

**Question**	**How often did you type on your phone while you were driving and when the car was moving?**	**Never**	**A few times**	**Most of the time**	**Always**			
Code	distract	0	1	2	3			
**Question**	**What's the most number of alcoholic drinks you've had prior to driving on any occasion?**	**#**						
Code	drinks	#						
**Question**	**Check off all the reasons you have not used a seat belt**	**Uncomfortable**	**Forgot**	**Don't think they help**	**Like freedom**	**Other**	**I always wear my seat belt**	
Code	seatbelt_reasons	0	1	2	3	4	5	
**Question**	**If other, state reasons**							
Code	seatbelt_other							
**Question**	**How often do your friends use their seat belt?**	**Never**	**Rarely**	**Most of the time**	**Always**			
Code		0	1	2	3			
**Question**	**How dangerous is it to not wear a seat belt?**	**Not at all**	**Somewhat**	**Very**	**Completely**			
Code	danger_seatbelt	0	1	2	3			
**Question**	**How much do you agree: I have complete control over whether I wear a seat belt.**	**Strongly Disagree**	**Disagree**	**Somewhat Agree**	**Mostly agree**	**Strongly Agree**		
Code	control_sb	0	1	2	3	4		
**Question**	**Check off all the reasons you have typed on your phone while driving?**	**I could not wait**	**I don't think it affects my driving**	**It was an emergency**	**It is fun**	**I get bored driving**	**I do not use my phone while driving**	**I do not drive**
Code	twd_reasons	0	1	2	3	4	5	6
**Question**	**How often do your friends type on their phones while driving?**	**Never**	**Rarely**	**Most of the time**	**Always**			
Code		0	1	2	3			
**Question**	**How dangerous is it to type on your phone while driving?**	**Not at all**	**Somewhat**	**Very**	**Completely**			
Code	danger_twd	0	1	2	3			
**Question**	**How much do you agree: I have complete control over whether I type on my phone while driving.**	**Strongly Disagree**	**Disagree**	**Somewhat Agree**	**Mostly agree**	**Strongly Agree**		
Code	control_twd	0	1	2	3	4		
**Question**	**Check off all the reasons you have driven a car after drinking alcohol?**	**I had no other opiton**	**I don't think it affects my driving**	**It's not a big deal**	**I am careful**	**It is fun**	**I do not drive after dirnking alcohol**	**I do not drive**
Code	dd_reasons	0	1	2	3	4	5	6
**Question**	**How often do your friends drive after drinking alcohol?**	**Never**	**Rarely**	**Most of the time**	**Always**			
Code	friends_dd	0	1	2	3			
**Question**	**How dangerous is it to drive soon after drinking more 3 or more alcoholic drinks?**	**Not at all**	**Somewhat**	**Very**	**Completely**			
Code	danger_dd	0	1	2	3			
**Question**	**How much do you agree: I have complete control over whether I type on my phone while driving.**	**Strongly Disagree**	**Disagree**	**Somewhat Agree**	**Mostly agree**	**Strongly Agree**		
Code	control_dd	0	1	2	3	4		
