# Supplementary material for: Effectiveness of a Text Message Intervention Promoting Seat Belt Use Among Young Adults: A Randomized Clinical Trial
Source: JAMA Netw Open. 2022 Sep 21;5(9):e2231616. doi: 10.1001/jamanetworkopen.2022.31616 (PMC9494210; doi:10.1001/jamanetworkopen.2022.31616)
Supplement: Supplement 2. — eTable 1. Univariate Associations Between Characteristics and Missing Web-Based Outcomes at 6 and 12 Weeks eTable 2. Seat Belt Cognitions at Baseline, 6 Weeks, and 12 Weeks [file jamanetwopen-e2231616-s002.pdf]

Supplemental Online Content

Suffoletto B, Pacella-LaBarbara ML, Huber J, Delgado MK, McDonald C. Effectiveness of a text message intervention promoting seat belt use among young adults: a randomized clinical trial. *JAMA Netw Open*. 2022;5(9):e2231616. doi:10.1001/jamanetworkopen.2022.31616

eTable 1. Univariate Associations Between Characteristics and Missing Web-Based Outcomes at 6 and 12 Weeks
eTable 2. Seat Belt Cognitions at Baseline, 6 Weeks, and 12 Weeks

This supplemental material has been provided by the authors to give readers additional information about their work.

© 2022 Suffoletto B et al. *JAMA Network Open.*

eTable 1. Univariate Associations Between Characteristics and Missing Web-Based Outcomes at 6 and 12 Weeks

	Missing	Complete	Univariate Odds ratio	P value
**6-weeks**, n	60	158		
Age in years, mean (SD)	22.0 (2.0)	21.4 (2.1)	1.2 1.0 to 1.3)	0.05
Non-White race	20 (33.3)	87 (55.1)	0.4 (0.2 to 0.8)	<0.01
Past year motor vehicle accident as driver	5 (8.3)	29 (18.4)	0.4 (0.2to 1.3)	0.1
SaVE intervention	28 (46.7)	82 (51.9)	0.8 (0.1 to 1.5)	0.5
% weeks with seat belt use, mean (SD)	45.2 (37.9)	50.2 (35.0)	0.7 (0.3 to 1.6)	0.4
**12-weeks,** n	78	140		
Age in years, mean (SD)	21.6 (1.9)	21.5 (2.1)	1.0 (0.9 to 1.2)	0.6
Non-White race	27 (34.6)	80 (57.1)	0.4 (0.2 to 0.7)	<0.01
Past year motor vehicle accident as driver	9 (11.5)	25 (17.9)	0.6 (0.3 to 1.4)	0.2
SaVE intervention	35 (44.9)	75 (53.6)	0.7 (0.4 to 1.2)	0.2
% weeks with seat belt use, mean (SD)	43.6 (35.8)	51.8 (35.5)	0.5 (0.2 to 1.2)	0.1

© 2022 Suffoletto B et al. *JAMA Network Open.*

eTable 2. Seat Belt Cognitions at Baseline, 6 Weeks, and 12 Weeks

	**Baseline**			**6-weeks**			**12-weeks**		
	**SaVE**	**Control**	**OR (95% CI)**	**SaVE**	**Control**	**OR (95% CI)**	**SaVE**	**Control**	**OR (95% CI)**
Total sample	110	108		81	74		74	65	
Friends use seat belts most of time	65 (59.1)	60 (55.6)	1.2 (0.7 to 2.0)	62 (76.5)	41 (55.4)	2.6 (1.3 to 5.2)	57 (77.0)	38 (58.5)	2.4 (1.1 to 5.0)
Believes completely dangerous to not wear a seat belt	59 (53.6)	65 (60.2)	0.8 (0.5 to 1.3)	55 (67.9)	52 (70.3)	0.9 (0.4 to 1.8)	51 (68.9)	44 (67.7)	1.1 (0.5 to 2.2)
Feels complete control over wearing seat belt	72 (65.5)	76 (70.4)	0.8 (0.5 to 1.4)	56 (69.1)	48 (63.9)	1.2 (0.5 to 2.4)	53 (71.6)	46 (70.8)	1.0 (0.5 to 2.2)

© 2022 Suffoletto B et al. *JAMA Network Open.*
