# Supplementary material for: Effectiveness of a Text Message Intervention Promoting Seat Belt Use Among Young Adults: A Randomized Clinical Trial
Source: JAMA Netw Open. 2022 Sep 21;5(9):e2231616. doi: 10.1001/jamanetworkopen.2022.31616 (PMC9494210; doi:10.1001/jamanetworkopen.2022.31616)
Supplement: Supplement 3. — Data Sharing Statement [file jamanetwopen-e2231616-s003.pdf]

Data Sharing Statement

Suffoletto. Effectiveness of a Text Message Intervention Promoting Seat Belt Use Among Young Adults. *JAMA Netw Open*. Published September 21, 2022.
doi:10.1001/jamanetworkopen.2022.31616

Data
Data available: Yes
Data types: Deidentified participant data
How to access data: Email: suffbp@stanford.edu for data
When available: With publication

Supporting Documents
Document types: None

Additional Information
Who can access the data: Researchers whose proposed use of the data has been approved
Types of analyses: For any purpose
Mechanisms of data availability: With investigator support
